# First-in-human phase 1 study of IT1208, a defucosylated humanized anti-CD4 depleting antibody, in patients with advanced solid tumors

**DOI:** 10.1186/s40425-019-0677-y

**Published:** 2019-07-24

**Authors:** Kohei Shitara, Satoshi Ueha, Shigeyuki Shichino, Hiroyasu Aoki, Haru Ogiwara, Tetsuya Nakatsura, Toshihiro Suzuki, Manami Shimomura, Toshiaki Yoshikawa, Kayoko Shoda, Shigehisa Kitano, Makiko Yamashita, Takayuki Nakayama, Akihiro Sato, Sakiko Kuroda, Masashi Wakabayashi, Shogo Nomura, Shoji Yokochi, Satoru Ito, Kouji Matsushima, Toshihiko Doi

**Affiliations:** 1 Department of Gastrointestinal Oncology, National Cancer Center Hospital East, Kashiwa, Japan; 2 Division of Molecular Regulation of Inflammatory and Immune Diseases, Research Institute for Biomedical Sciences, Tokyo University of Science, 2669 Yamazaki, Noda, Chiba 278-0022 Japan; 3 Department of Molecular Preventive Medicine, Graduate School of Medicine, The University of Tokyo, Bunkyo-ku, Tokyo Japan; 4 Division of Cancer Immunetherapy, National Cancer Center, Exploratory Oncology Research and Clinical Trial Center (EPOC), Chuo-ku, Tokyo Japan; 5 Department of Experimental Therapeutics, National Cancer Center Hospital, Chuo-ku, Tokyo Japan; 6 Clinical Research Support Office, National Cancer Center Hospital East, Kashiwa, Chiba Japan; 7 IDAC Theranostics Inc., Bunkyo-ku, Tokyo Japan; 8 Department of Experimental Therapeutics, National Cancer Center Hospital East, 6-5-1 Kashiwanoha, Kashiwa, Chiba 277-8577 Japan

**Keywords:** Anti-CD4 antibody, CD4^+^ T cells, CD8^+^ T cells, Immunotherapy

## Abstract

**Background:**

Transient CD4^+^ T cell depletion led to the proliferation of tumor-specific CD8^+^ T cells in the draining lymph node and increased infiltration of PD-1^+^CD8^+^ T cells into the tumor, which resulted in strong anti-tumor effects in tumor-bearing mice. This is a first-in-human study of IT1208, a defucosylated humanized anti-CD4 monoclonal antibody, engineered to exert potent antibody-dependent cellular cytotoxicity.

**Methods:**

Patients with advanced solid tumors were treated with intravenous IT1208 at doses of 0.1 or 1.0 mg/kg. The first patient in each cohort received a single administration, and the other patients received two administrations of IT1208 on days 1 and 8.

**Results:**

Eleven patients were enrolled in the 0.1 mg/kg (*n* = 4) and 1.0 mg/kg cohorts (*n* = 7). Grade 1 or 2 infusion-related reactions was observed in all patients. Decreased CD4^+^ T cells in peripheral blood due to IT1208 were observed in all patients and especially in those receiving two administrations of 1.0 mg/kg. CD8^+^ T cells increased on day 29 compared with baseline in most patients, resulting in remarkably decreased CD4/8 ratios. One microsatellite-stable colon cancer patient achieved durable partial response showing increased infiltration of both CD4^+^ and CD8^+^ T cells into tumors after IT1208 administration. Moreover, transcriptomic profiling of the liver metastasis of the patient revealed upregulation of the expression of interferon-stimulated genes, T cell activation-related genes, and antigen presentation-related genes after IT1208 administration. Two additional patients with gastric or esophageal cancer achieved stable disease lasting at least 3 months.

**Conclusions:**

IT1208 monotherapy successfully depleted CD4^+^ T cells with a manageable safety profile and encouraging preliminary efficacy signals, which warrants further investigations, especially in combination with immune checkpoint inhibitors.

**Electronic supplementary material:**

The online version of this article (10.1186/s40425-019-0677-y) contains supplementary material, which is available to authorized users.

## Introduction

Immune checkpoint inhibitors such as anti-cytotoxic T-lymphocyte-associated antigen-4 and anti-programmed cell death-1 (PD-1) monoclonal antibody (mAb) agents or their combinations have improved outcomes of various cancers [1–7]. However, many patients fail to achieve clinical benefit, highlighting the importance of additional treatment to overcome resistance. One of the possible reasons for treatment failure with PD-1 blockade is the presence of immune suppression through immune checkpoints other than the PD-1/programmed cell death ligand-1 (PD-L1) axis regulating lymphocyte activation and expansion or through immune suppressive cells including forkhead box P3 (Foxp3)^+^ CD25^+^ regulatory T cells (Tregs), T helper 2 (Th2) cells, myeloid-derived suppressor cells (MDSCs), tumor-associated macrophages, and plasmacytoid dendritic cells (pDCs) [8–13].

Several reports suggested that depletion of CD4^+^ cells, including Tregs, Th2 cells, and a subpopulation of MDSCs and pDCs, results in strong antitumor effects in mouse models due to the enhancement of cytotoxic T-lymphocyte responses [14–16]. Previously, we showed that administration of the anti-CD4 mAb had strong antitumor effects superior to those elicited by CD25^+^ Treg depletion or other immune checkpoint mAbs in B16F10, Colon 26, or Lewis lung carcinoma subcutaneous tumor models, which were completely reversed by CD8^+^ cell depletion [17]. CD4^+^ cell depletion led to the proliferation of tumor-specific CD8^+^ T cells in the draining lymph node (dLN) and increased infiltration of PD-1^+^ CD8^+^ T cells into the tumor with a shift toward type I immunity within the tumor [17]. The augmentation of antitumor CD8^+^ T-cell responses appeared as increasing numbers of CD8^+^ T-cell clones that overlapped among the tumor, dLN, and peripheral blood repertoires [18]. Further, combination treatment with the anti-CD4 mAb and immune checkpoint mAbs, particularly anti-PD-1 or anti-PD-L1 mAbs, synergistically suppressed tumor growth and greatly prolonged survival [17].

IT1208 (IDAC Theranostics, Inc., Tokyo, Japan US8399621) is a humanized anti-CD4 immunoglobulin G1 mAb with a defucosylated Fc region, which markedly enhances antibody-dependent cellular cytotoxicity (ADCC) [19]. The present study is a first-in-human, phase I, open-label, dose-escalation study conducted to assess the safety, pharmacokinetics, pharmacodynamics, and immunological mechanisms of action of IT1208 when administered as monotherapy to patients with advanced solid tumors.

## Materials and methods

### Study design

The primary objective of this study was to evaluate safety and to determine the maximum tolerated dose (MTD) and recommended dose (RD) of IT1208. Secondary objectives included assessing incidences of adverse events, pharmacokinetics of IT1208, serum CD4^+^ T-cell counts as a pharmacodynamic marker of IT1208, as well as objective tumor response and progression-free survival (PFS). Exploratory biomarker analysis included histological and transcriptomic analyses of the tumor to assess cellular and molecular effects of IT1208. Immune phenotyping and T-cell receptor (TCR) repertoire analysis were also conducted (Additional file 1: Figure S1). The study was conducted in accordance with the Declaration of Helsinki and Good Clinical Practice Guidelines, following approval by the ethics board in each institution. The study protocol was registered at the University Hospital Medical Information Network Clinical Trials Registry (protocol ID UMIN000026564).

### Patient eligibility

Criteria for patient enrollment in the study included (1) advanced or metastatic solid tumors resistant to standard therapy or without available standard therapy, (2) an Eastern Cooperative Oncology Group performance status of 0 or 1, (3) adequate bone marrow reserve (neutrophil count of ≥1500/mm^3^, hemoglobin level of ≥9.0 g/dl, platelet count of ≥100,000/mm^3^, and absolute lymphocyte count ≥800/mm^3^), and (4) adequate hepatic and renal function. Patients who had previously received immune checkpoint inhibitors such as anti-PD1 or anti-PD-L1 mAb were eligible if the treatment had been discontinued at least 4 weeks prior to study. Major exclusion criteria included (1) a history of chemotherapy or radiotherapy within the previous 2 weeks, (2) patients with acquired immunodeficiency syndrome or with active autoimmune diseases requiring steroid (glucocorticoid) treatment or other immunosuppressants. Treatments, such as hormone supplementation for hypothyroidism, were not included in the exclusion criteria, (3) patients with active infectious diseases requiring systemic treatment, (4) history of tuberculosis, (5) positive test results for hepatitis B surface antigen, hepatitis B virus antibody, or hepatitis C virus antibody, or (6) the presence of serious comorbidity. All patients provided written informed consent for participation in the study.

### Drug administration and dose-escalation procedure

Eligible patients were treated with an intravenous infusion of IT1208 at planned doses of 0.1, 1.0, and 3.0 mg/kg. The first patient in each cohort was treated with one dose of IT1208 on day 1, and other patients received two doses of IT1208 on days 1 and 8 followed by safety and efficacy assessment until disease progression or the development of intolerable toxicity. Dose-limiting toxicities (DLTs) were evaluated during the DLT evaluation period from the initial dose to day 29. DLTs were defined as any of the following toxicities judged to be caused due to IT1208: grade 4 neutropenia; grade 4 thrombocytopenia or thrombocytopenia requiring transfusions; grade ≥ 3 febrile neutropenia; uncontrollable nonhematologic toxicity of grade ≥ 3 despite maximal supportive care, excluding manageable grade 3 infusion-related reactions; and toxicities that required treatment delay of ≥3 days or discontinuation of the planned day 8 infusion.

If no DLTs were observed in the first subject of the one-dose group, administration of the drug at the same dose was started in the two-dose group. If no DLTs were observed in the first three subjects, the next dosage level was opened. If DLTs were observed in one out of the first three subjects, up to six subjects were planned to be added at the same dose level to evaluate DLTs. The MTD was defined as the highest dosage level that does not lead to DLTs in one or none out of six subjects. The RD for the next study was defined as one dose level below the MTD or the maximum dose level judged to be tolerable. Even if dose escalation to the next dosage level was possible, if the clinical trial-coordinating committee determines that additional cases were required for further evaluation, such as pharmacokinetics, subjects could be added at that dose level. The data center of the Clinical Research Support Office in the National Cancer Center Hospital East, Japan (NCCHE-OCRS) confirmed patient eligibility, and the dose level was then assigned. Data collection, data analysis, and data interpretation were also performed by the NCCHE-OCRS (study number; EPOC 1601).

### Assessment

Adverse events were evaluated until day 92 or the beginning of subsequent treatment per the National Cancer Institute Common Terminology Criteria for Adverse Events (CTCAE version 4.0). Tumor measurements were obtained using computed tomography at baseline every 4 weeks until disease progression or the beginning of subsequent treatment. Since this is FIH trial with only one or two administration of IT1208, we applied this relatively frequent tumor measurement. Tumor response was evaluated per the Response Evaluation Criteria in Solid Tumors (RECIST, v1.1), and PFS of each patient was assessed. PFS was defined as the time from the date of registration until the date of disease progression or the date of death from any cause, whichever earliest one among the following events.

Serum samples were obtained at various time points for pharmacokinetic, cytokine, and chemokine analysis. Whole blood was collected and processed into peripheral blood mononuclear cells (PBMCs) and frozen for subsequent analysis using flow cytometry and TCR repertoire analysis. Tumor biopsy was conducted before the study treatment and immediately after DLT evaluation period (on day 29) for multicolor immunohistochemistry and transcriptomic analyses (see Additional file 2: Appendix Materials and Methods).

### Pharmacokinetic analysis

The pharmacokinetic parameters were calculated via noncompartment analysis using an analytical approach similar to the intravenous infusion model generated by WinNonlin Professional version 6.4 (Certara USA, Princeton, NJ, USA). The maximum concentration (*C*_max_) values were obtained from measured values. The apparent elimination half-life (*t*_1/2z_) was obtained in the terminal phase (see Additional file 1: Appendix Materials and Methods).

### Evaluation of biopsy tumor tissue

Three-micrometer sections of formalin-fixed, paraffin-embedded tumor biopsies were evaluated with hematoxylin&eosin (H&E) and multiplex fluorescence immunohistochemical (mFIHC) analysis using an Opal kit. Images were captured using Vectra ver.3 (PerkinElmer, Hopkinton, MA, USA), and an image-analysis program (Inform) was used to detect immune cells with specific phenotypes in the tumor or stroma areas. The number of infiltrating immune cells was normalized with the tumor or stroma areas and quantified as the density of cells per mm^2^.

### Statistical analysis

Patient characteristics, safety data, antitumor activity were summarized descriptively. Quantitative data were summarized descriptive statistics such as median, range, mean and standard deviation, etc. Statistical analyses were performed using SAS Release version 9.4 (SAS Institute, Cary, NC, USA).

## Results

### Patient characteristics

Eleven patients were enrolled in the study between April 2017 and February 2018 (Table 1). Four and seven patients were assigned to dose groups of 0.1 mg/kg (level 1) and 1.0 mg/kg (level 2), respectively, and comprised those with gastric or gastro-esophageal cancer (*n* = 6), colorectal cancer (*n* = 3), esophageal cancer (*n* = 1), and pancreatic cancer (*n* = 1, also with simultaneous colon cancer). Median lines of previous chemotherapy were 5 (range 2–11). Four patients previously received anti-PD1/PD-L1 inhibitors; Cases 1, 2, 9 with gastric cancer and Case 6 with colorectal cancer had been previously treated with anti-PD1/PDL1 targeting treatment. Median duration of last dose of anti-PD1/PDL1 to study enrollment was 6.3 months (range 1.6 to 15.9 months).

**Table 1:** Baseline Characteristics of the 11 Patients Enrolled in the Study

Characteristic		Patients	
		*n*	%
Age, years	Median (range)	65 (35–79)	–
Sex	Male	8	73
	Female	3	27
ECOG performance status	0	11	100
Cancer types	Gastric or gastro-esophageal	6	55
	Colorectal	4	36
	Esophageal	1	9
	Pancreas	1	9
Microsatellite instability status	Microsatellite stable	9	82
	Unknown	2	18
Previous treatment line	Median (range)	5 (2–11)	–
Previous anti-PD1/PDL1	Yes	4	36
Metastatic sites	Lymph node	8	73
	Lung	5	45
	Liver	4	36

*ECOG* Eastern Cooperative Oncology Group

### Tolerability and adverse events

All patients completed the planned one or two administrations of IT1208. Initially, IT1208 was infused over 2 h. Patients receiving 0.1 mg/kg did not receive any premedication. Because interruption of administration due to infusion-related reactions occurred in the first three patients receiving two doses of 1.0 mg/kg, infusion of IT1208 was prolonged from 2 to 4 h, and 100 mg hydrocortisone was also injected intravenously before each IT1208 administration, and three additional patients were enrolled in the 1.0 mg/kg group (six patients in total). Although no DLT was observed at 1.0 mg/kg, we did not further escalate the dose because clear depletion of CD4^+^ T cells in PBMCs was seen. Therefore, MTD was not confirmed in this study, and the RD for further investigation was set at 1.0 mg/kg.

The only IT1208-related adverse events were grade 1 or 2 infusion-related reactions, which were observed in all patients. Patients receiving 0.1 mg/kg were associated with grade 1 infusion-related reactions with the primary symptom of fever (Additional file 3: Table S1). All six patients receiving administrations of 1.0 mg/kg experienced grade 2 infusion-related reactions with common symptoms including chills (*n* = 6, 100%), fever (*n* = 4, 67%), nausea (*n* = 3, 50%), and vomiting (*n* = 3, 50%). One patient experienced a transient decline in oxygen saturation and hypotension (Case 7). All symptoms recovered after transient interruption of IT1208 administration, and the remaining IT1208 administrations were completed within a day in all patients. No other treatment-related adverse events including immune-mediated or apparent infections were observed. No patients experienced severe adverse events.

### Pharmacokinetics and pharmacodynamics

The maximum plasma concentration (*C*_max_) of IT1208 at a dose of 0.1 mg/kg after one or two administrations was 0.255 ± 0.0838 μg/mL (average ± standard deviation) and decreased to less than the detection threshold (0.03 μg/mL) at 4 h after the end of infusion (Additional file 4: Figure S2). The concentration of IT1208 after one or two administrations at 1.0 mg/kg was 16.2 ± 2.32 μg/mL and decreased to less than the detection threshold at 72 h after the end of infusion, with a *t*_1/2z_ of 17.5 ± 2.54 h. Exposure was dose proportional, and no accumulation of IT1208 was observed at both dose levels before the second infusion on day 8.

Serum cytokines including IL-6, IL-8, and TNF-alpha were investigated in cases 9–11. Elevated levels of IL-6, IL-8, and TNF-alpha during each infusion were observed, possibly causing mild cytokine-release syndromes with fever, chills, and hypotension (Additional file 5: Figure S3).

**Fig. 1:**
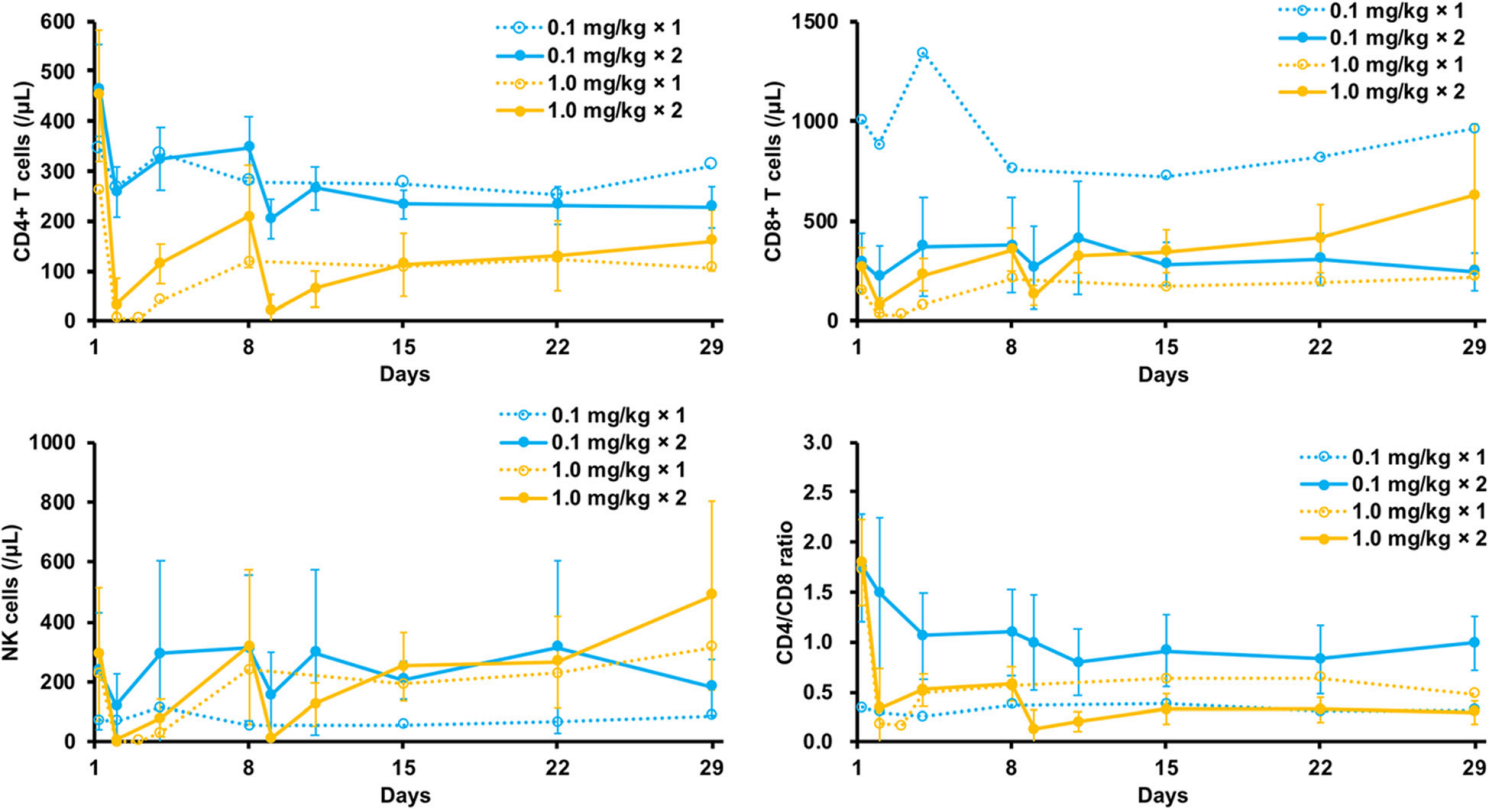
Mean peripheral counts of CD4^+^ and CD8^+^ T cells and NK cells. Mean counts of CD4^+^ and CD8^+^ T cells and NK cells in PBMCs at each dose level are shown. Decreased CD4^+^ T-cell count due to IT1208 was more apparent in patients receiving 1.0 mg/kg rather than 0.1 mg/kg. CD8^+^ T-cell counts and NK-cell counts were also decreased immediately after IT1208 administration, but CD8^+^ T-cell counts subsequently increased until day 29 and surpassed the baseline level, which resulted in remarkably decreased CD4/8 ratios

Decreased CD4^+^ T-cell counts in PBMCs due to IT1208 were observed in all patients, especially in patients receiving two administrations of 1.0 mg/kg, who experienced a reduction of CD4^+^ T cell count from a median 395/μL at baseline to 3.5/μL at nadir (Fig. 1, Additional file 6: Figure S4). Relative change of CD4^+^ T cell counts from baseline to nadir were larger in 1.0 mg/kg than 0.1 mg/kg (median − 98.7% vs. -54.4%, Wilcoxon rank sum test *p* = 0.0107). CD8^+^ T cell counts were also decreased immediately after IT1208 administration but then increased until day 29 and surpassed baseline counts in most patients, which resulted in remarkably decreased CD4/8 ratios. Relative changes of CD8^+^ T cell counts, CD4/8 ratios, or NK cells from baseline to those on day 29 was 118% in 1.0 mg/kg and − 8.9% in 0.1 mg/kg (*p* = 0.0182), − 84.9% in 1.0 mg/kg and − 36.6% in 0.1 mg/kg (*p* = 0.0107), or − 37.0% in 1.0 mg/kg and 4.1% in 0.1 mg/kg (*p* = 0.0726).

NK cell counts were also decreased immediately after IT1208 administration but increased subsequently. Other leukocyte populations, including monocytes and plasmacytoid DCs, were not significantly changed during observation period, except that minor NK cells and mDCs decreased with statistical significance in 1.0 mg/kg at day 29 (Additional file 7: Figure S5).

**Fig. 2:**
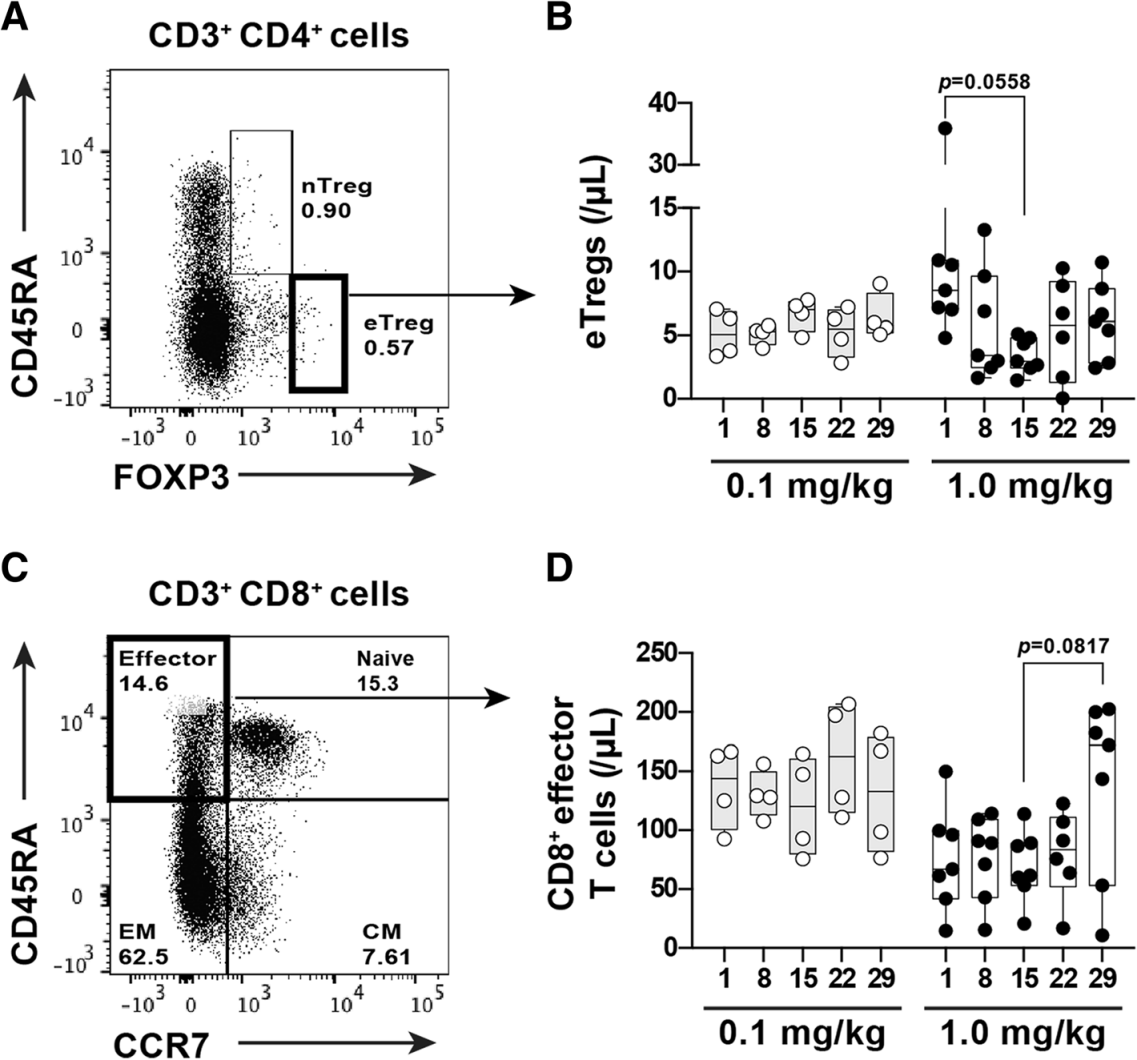
Kinetics of eTregs and effector CD8^+^ T cells in PBMCs after IT1208 administration **a** and **c**. The lymphocyte gating strategy for identification of eTregs (**a**) and effector CD8^+^ T cells (**c**) is indicated. The eTregs were identified as CD4^+^CD45RA^−^FoxP3^high^ and effector CD8^+^ T cells were identified as CD8^+^CD45RA^+^CCR7^−^, respectively. **b** and **d**. Changes in eTregs (**b**) and effector CD8^+^ T cells (**d**), after infusion with 0.1 mg/kg (gray bar and circle point) and 1.0 mg/kg (white bar and square point) IT1208. In patients receiving 1.0 mg/kg IT1208, the number of eTregs tended to decrease (**b**; *n* = 7, Wilcoxon’s rank sum test, *p* = 0.0558); in contrast, effector CD8^+^ T cells tended to increase (**d**; *n* = 7, *p* = 0.0817); this did not occur at 0.1 mg/kg. The cell numbers are presented as Box and Whiskers plots. One-way ANOVA (using Prism7) was used to perform multiple comparisons of the means of cell numbers among the time periods

Since the antitumor effects of anti-CD4 depleting antibodies are associated with the depletion of Tregs in murine models, we evaluated the kinetics of the FoxP3^hi^CD45RA^−^ Treg population (Fig. 2a), known as the eTreg subset and associated with augmentation strong immunosuppressive functions, in PBMCs after IT1208 administration. The number of eTregs transiently decreased in the 1.0 mg/kg group with the lowest count at day 15 after IT1208 administration and recovered subsequently (Fig. 2b). Moreover, following the decrease in eTregs, the population of CD45RA^hi^CCR7^−^ effector CD8^+^ T cells tended to increase in later phases (Fig. 2c and d).

### Antitumor activity and tumor biomarkers

**Fig. 3:**
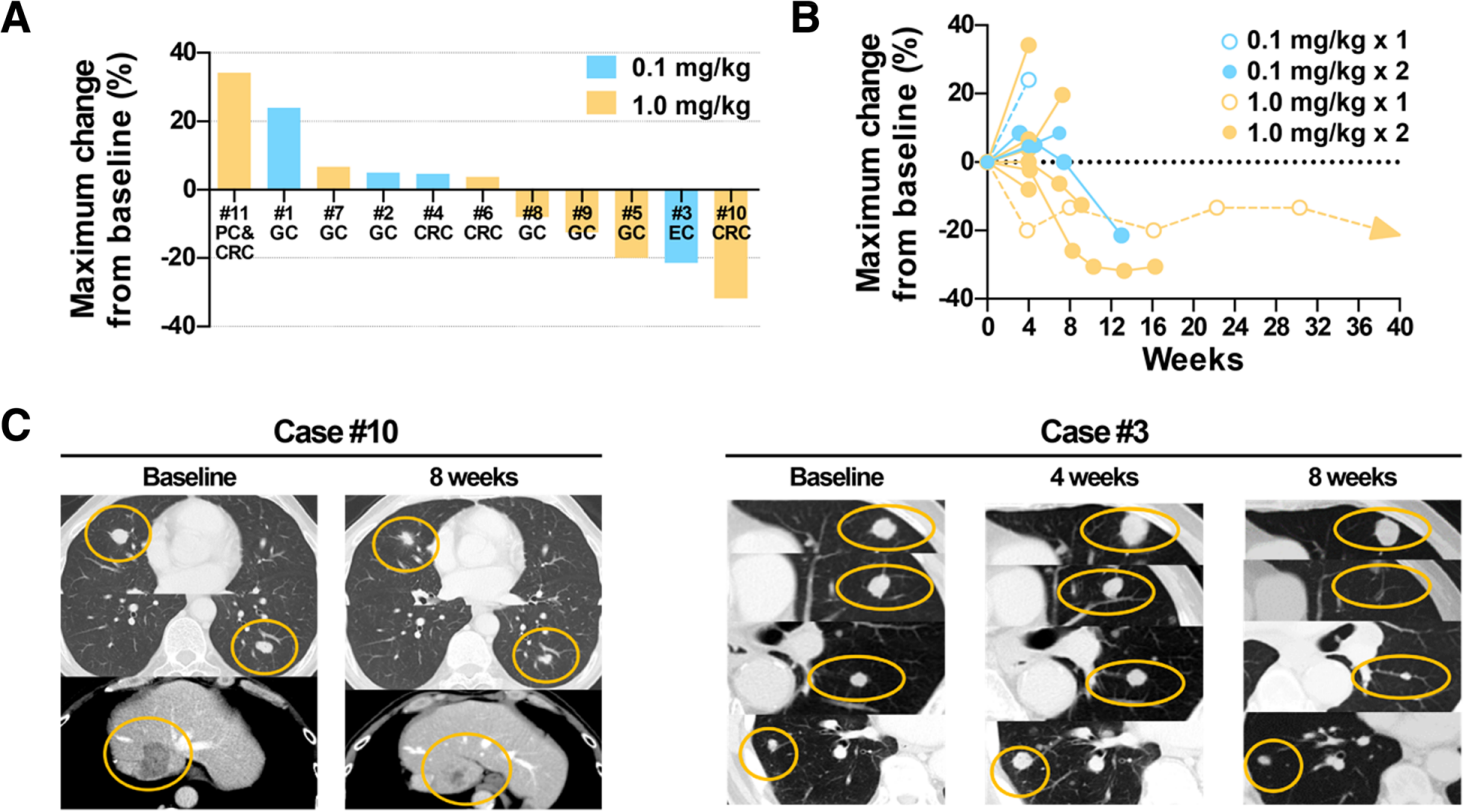
Antitumor Activity of IT1208. **a** Waterfall plot of maximum tumor change from baseline in each patient. GC, gastric cancer; CRC, colorectal cancer; EC, esophageal cancer; PC, pancreatic cancer. **b** Spider plot of each patient. **c** Representative case with antitumor response. Case 10 had colorectal cancer with liver and lung metastases. This patient had been previously treated with fluoropyrimidines, oxaliplatin, irinotecan, bevacizumab, and panitumumab and then progressed. The patient experienced a PR (32% shrinkage of target lesions) at 2 months after IT1208 treatment. Tumor responses were maintained for more than 3 months at the data cutoff. Case 3 with esophageal squamous cell carcinoma also showed tumor shrinkage (21%), being disease free for 3.1 months

The best objective response was partial response (PR) in one patient (Case 10) with microsatellite-stable colorectal cancer and lung and liver metastases receiving two doses of IT1208 1.0 mg/kg (Fig. 3a-c). This patient had been previously treated with first-line treatment containing oxaliplatin, capecitabine, and bevacizumab as well as second-line treatment with irinotecan and panitumumab. Tumor response by IT1208 was maintained until more than 3 months at the data cut off. Seven patients showed stable disease, and two had stable disease lasting more than 3 months (Case 3 with esophageal squamous cell carcinoma with PFS of 3.1 months, and Case 5 with gastric cancer with PFS > 8.9 months). Overall, five patients had some degree of tumor shrinkage (Fig. 3). CT images indicated that the preselected target lesions shrunk in cases 10 and 3 at 8 weeks compared to baseline (Fig. 3c).

**Fig. 4:**
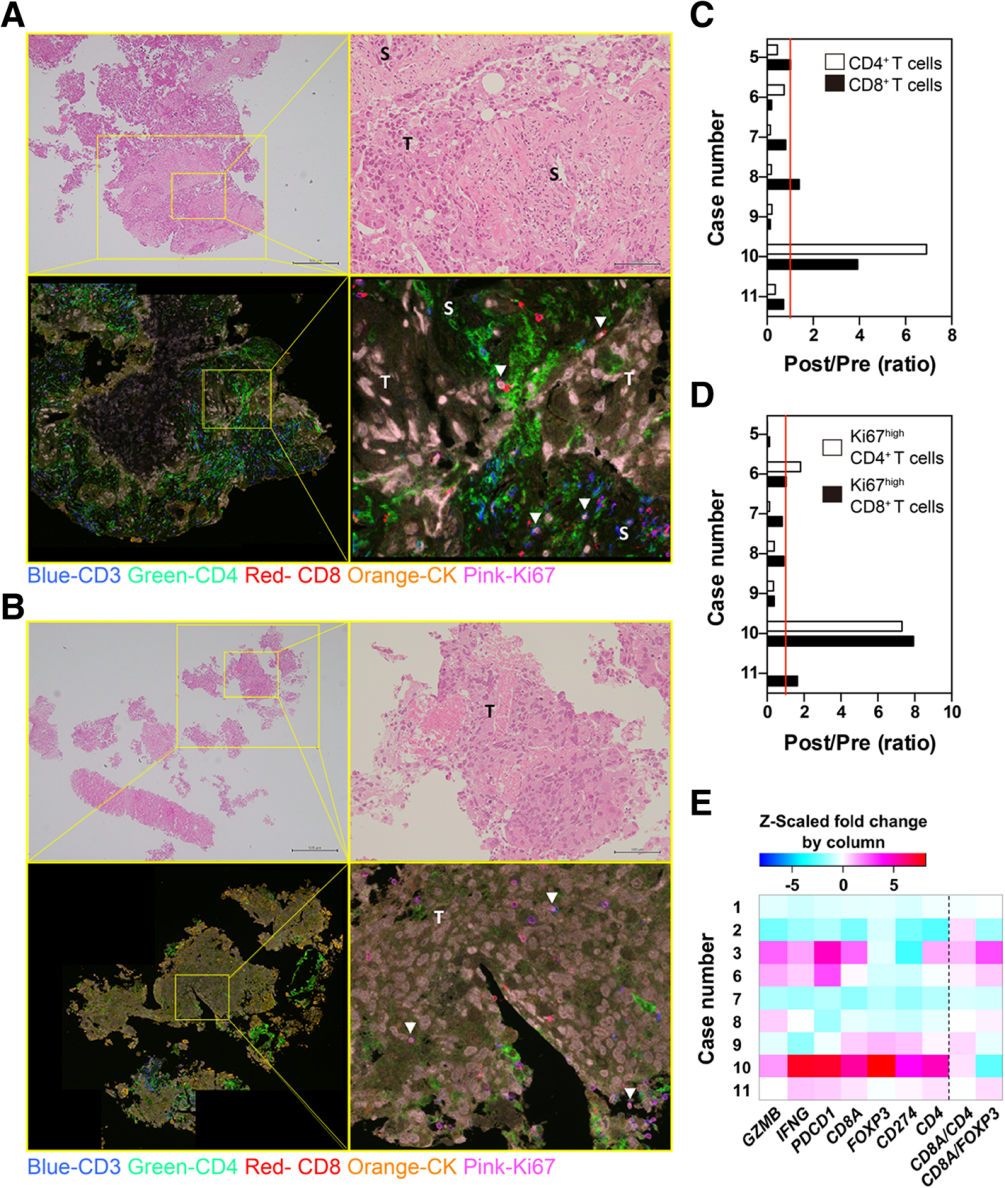
Evaluation of tumor microenvironment after IT1208 administration. **a** and **b**. Representative images of biopsy specimens stained using mFIHC pre (**a**) and post (**b**) IT1208 treatment in Case 10. Upper photographs show H&E staining with low (left) and high (right) magnification (scale bars: 500 or 100 μm, respectively). Activation of T cells was determined by visualizing nuclear Ki67 (pink) expression in each subset. CD4^+^ or CD8^+^ T cells were detected by visualizing CD3 (blue) and CD4 (green) double-positive or CD3 (blue) and CD8 (red) double-positive cells, respectively. Tumor (T) and stroma (S) were determined as CK-positive (orange) and -negative areas, respectively. White arrowheads (▽) in lower right images indicate CD3^+^CD8^+^Ki67^high^ cells. **c** and **d**. Changes in CD3^+^CD8^+^ or CD3^+^CD4^+^ T cells (**c**), and the Ki67^high^ population (**d**) after IT1208 treatment. The density of each T-cell subset was evaluated (Appendix Table A2), and the ratio of changes between pre and post IT1208 treatment in each patient is shown. **e**. Heat map of the changes in gene expression in the tumor biopsies following IT1208 treatment. Columns represent fold changes in expression of each gene or the relative value of gene expression, while rows represent case number. The *Z*-scaled fold changes by column are shown

Next, we assessed the effect of IT1208 on the tumor microenvironment by evaluating lymphocyte infiltration and gene expression. Figures 4a and b show representative images of H&E and mFIHC staining of Case 10. The density of lymphocyte subsets infiltrating into tumor tissue was quantified in PanCK-positive tumor areas. The density of CD3^+^CD4^+^ T cells in the tumor decreased in six of seven patients receiving 1.0 mg/kg IT1208 (Fig. 4c and Additional file 8: Table S2), while case 10 with PR showed a dramatic increase in the density of both CD3^+^CD8^+^ and CD3^+^CD4^+^ T cells in the tumor area (Fig. 4c). Moreover, the density of Ki67^+^-activated T cells also increased after IT1208 administration in case 10 (Fig. 4d). We also evaluated CD204^+^ CD4^+^ macrophages and found no significant change in the CD204^+^ CD4^+^ area in the tumor of patients receiving two doses of 1.0 mg/kg IT1208 (Additional file 9: Figure S6).

Consistently, the tumors in cases 10 and 3 showed remarkable upregulation of antitumorigenic genes including *IFNG*, *GZMB*, and *CD8A* and interferon-related and T-cell activation-related genes including *CD40*, *STAT1*, and *IRF1* (Fig. 4e and Fig. A5). Of note, expression of MHC class I genes (*HLA-E*, *HLA-F*, and *HLA-G*) increased in the tumors in Cases 10 and 3 after IT1208 administration (Additional file 10: Figure S7).

**Fig. 5:**
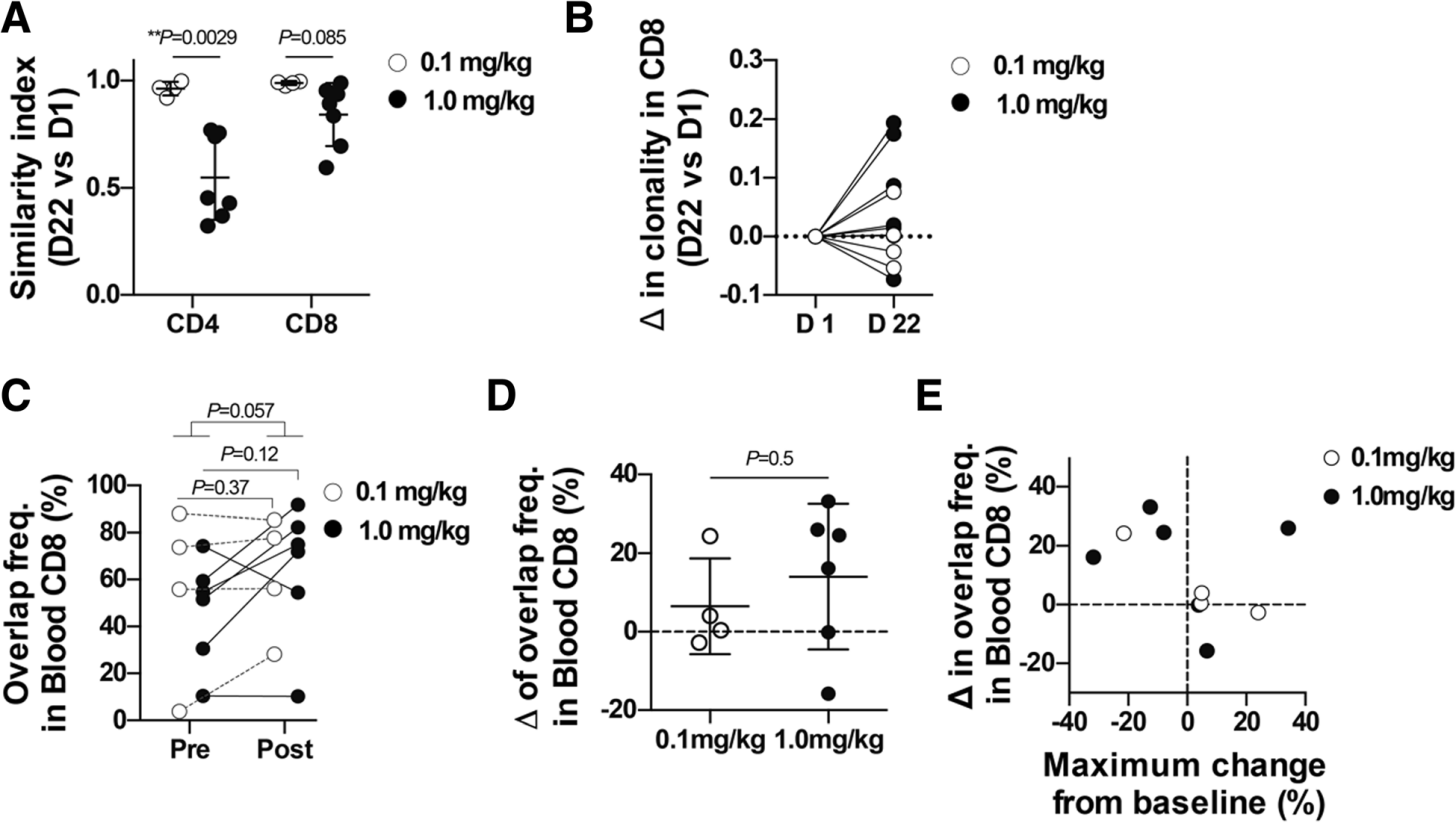
Effects of IT1208 treatment on TCR repertoire. TCR repertoires of the blood CD4^+^ and CD8^+^ T cells and the tumor tissues were analyzed using next-generation sequencing. **a**. Changes in the TCR repertoires of the blood CD4^+^ and CD8^+^ T cells following IT1208 treatment are shown as R similarity index. Lower similarity index indicates greater variation in TCR repertoire following IT1208 treatment. *P* values obtained by an unpaired, two-tailed Student’s *t* test. **b**. Changes in 1-Pielou clonality index of TCR repertoire in the blood CD8^+^ T cells. **c**. The sum total frequency of tumor-blood overlapping clones in blood CD8^+^ T cells. The clones overlapping between blood and tumor were identified at pre- and post-treatment, and the sum total frequency of these overlapping clones is plotted. **d**. Comparison of the changes in sum total frequency of Blood-Tumor overlapping clones between 0.1 mg/kg and 1 mg/kg group in blood CD8^+^ T cells. **e**. Scatter plot of the changes in the frequency of overlapping CD8^+^ T-cell clones in the blood and maximum change from baseline. Each dot represents individual patients. White and black represent 0.1 and 1.0 mg/kg IT1208 treatment, respectively. **a** and **d** Unpaired, two-tailed Student’s *t* test. (C) Paired, two-tailed Student’s *t* test for comparison within group, unpaired, two-tailed Student’s *t* test for comparison between groups. **, *P* < 0.01

Similarity index comparing TCR repertoires in blood T cells before and after IT1208 administration was low in the 1.0 mg/kg group, particularly in CD4^+^ T cells (Fig. 5a; Additional file 11: Table S3, Additional file 12: Table S4,). Clonality of blood CD8^+^ T cells increased in all patients receiving two doses of 1.0 mg/kg (Fig. 5b). We also examined the overlap of TCR repertoires between the PBMCs and the tumor biopsies, which are presumably enriched for clones associated with antitumor T-cell responses. Sum total frequency of overlapping clones in the blood CD8^+^ T cells tended to increase after IT1208 administration, particularly in patients receiving 1.0 mg/kg (Fig. 5c, d), and four of five patients with more than 10% increased frequency of overlap achieved tumor shrinkage (Fig. 5e).

## Discussion

We evaluated the mode of action, tolerated dose, and pharmacokinetics of IT1208 as an anti-CD4 depleting mAb in patients with advanced solid tumors. To the best of our knowledge, this is the first study evaluating the effect of depletion of CD4^+^ cells in solid tumors. Depletion of CD4^+^ T cells was clearly confirmed in PBMCs, which was also associated with an increased fraction of CD8^+^ T cells and decreased CD4/CD8 ratios following IT1208 administration. Depletion of CD4^+^ T cells appeared to also be dose dependent; a higher dose showed stronger CD4^+^ T-cell depletion. Transient decrease in nontarget CD8^+^ T cells and NK cells might have been induced by cytokines including IL-6 and TNF-alpha released from activated NK cells, which lyse antibody-bound CD4^+^ T cells through ADCC. The increase in cytokines might have also contributed to mild infusion-related reactions. Transient depletion of CD4^+^ T cells did not lead to nosocomial infections, although follow-up periods in this study were relatively short. Although there were no DLTs, we did not escalate the dose above 1.0 mg/kg, considering that depletion of CD4^+^ T cells in PBMC was seen.

In the present study, one PR was observed in MSS colorectal cancer, in which PD-1 blockade is usually not active [20]. This patient also showed increasing Ki67^+^ CD4^+^ and CD8^+^ T cells along with the upregulation of antitumor gene expression localized to the tumor after IT1208 administration. This is consistent with our previous experience in a preclinical study, which showed that transient depletion of CD4^+^ cells expands CD8^+^ T cells and shifts the tumor microenvironment toward type I immunity [17]. Transient CD4 depletion from the circulation but not continuous CD4 depletion from the tumor might be sufficient to exert antitumor T-cell responses due to IT1208 monotherapy. Meanwhile, change of CD4^+^ T cells in the local tumor were inconsistent across the enrolled patients. Although this observation might be due to intra-tumor heterogeneity or timing of biopsy, further research is necessary to clarify the effect of IT1208 on local immunity.

TCR repertoire analyses revealed that IT1208 not only reduced the number of CD4^+^ T cells but also altered the repertoire (or antigen reactivity) dose dependently. Increase of clonality in blood CD8^+^ TCR repertoires, which often occur during antigen-specific T-cell responses, was also dose dependent although the antigen reactivity was not addressed in the present study. Increase in tumor-blood overlapping clones in the blood CD8^+^ TCR repertoires in the 1.0 mg/kg group (Cases 8–11) and a responder in the 0.1 mg/kg group (Case 3) is consistent with our preclinical study [18], suggesting the augmentation of tumor-specific CD8^+^ T-cell responses. Our transcriptomic analysis identified upregulated expression of MHC class I, which present tumor antigens to CD8^+^ T cells and mediate CD8^+^ T cell-dependent antitumor effects [21], in Cases 3, 4, and 10. Considering that both tumor-blood overlapping clones and MHC class I gene expression increased in the IT1208 responders (Cases 3 and 10), the increase of tumor-blood overlapping CD8^+^ TCR clones and MHC class I gene expression may reflect the mode of action of CD4 depletion [17] and may be a response marker to IT1208 treatment.

Interestingly, decrease in CD4^+^ T cells or CD4 gene expression in the tumor was not associated with tumor shrinkage in some patients. Therefore, additional treatment might be necessary to improve outcomes further. The decrease in eTregs and increase in effector CD8^+^ T cells might form a rationale for combinations of IT1208 with additional therapies such as anti-PD-1 or PD-L1 inhibitors. Several studies have suggested that presence of immune suppressive cells could be one of the reasons for resistance to checkpoint inhibitors [8–13]. In contrast, our previous preclinical study using CD4 depletion and PD-1 blockade showed robust synergistic efficacy [17], which warrants further investigation of IT1208 in combination with immune checkpoint inhibitors.

Although there is a risk of immunodeficiency following the depletion of CD4^+^ T cells, current protocol did not completely and continuously deplete CD4^+^ helper T cells even at two injections of 1.0 mg/kg. The residual CD4^+^ T cells might contribute to basal humoral immunity during the observation period. In addition, there are several humoral and cellular immunological memories, such as Ag specific immunoglobulins, long-lived plasma cells, and memory B and CD8^+^ T cells, all of which are maintained independently of CD4^+^ T cells. These immunological memory and innate immune cells might also protect the patients from infection during our observation period. However, we should take caution because the depletion of CD4^+^ T cells decreases humoral immune responses to inexperienced virus infection, such as seasonal influenza infection and so on.

The major limitation of the present study was its small sample size with various cancer types as a first-in-human phase 1 study; nevertheless, our findings suggest feasibility of IT1208 use among these populations. We infused IT1208 using only one or two administrations, and thus, long-term safety could not be evaluated. As mentioned previously, we did not escalate the dose above 1.0 mg/kg, thus MTD could not be defined in this study. Moreover, optimal dose and treatment schedule, especially in combination with immune checkpoint inhibitors, should be evaluated in a separate study. Requirement for immunological background of the patients, such as the presence of pre-existing anti-tumor CD8^+^ T cells, and sensitivity of tumor type should also be investigated in the future study.

## Conclusion

We have shown that IT1208 monotherapy successfully depleted CD4^+^ T cells with a manageable safety profile and encouraging preliminary efficacy signals, which warrants further investigations, especially in combinations with immune checkpoint inhibitors.

## Additional files

Additional file 1: **Figure S1.** Flowchart of blood sample analyses in clinical trial and translational research. (DOCX 226 kb)

Additional file 2: Appendix Materials and Methods. (ZIP 358 kb)

Additional file 3: **Table S1.** IT1208-related adverse events. (DOCX 13 kb)

Additional file 4: **Figure S2.** Pharmacokinetics of IT1208 and serum level of cytokines. (DOCX 128 kb)

Additional file 5: **Figure S3.** Serum level of cytokines. (DOCX 99 kb)

Additional file 6: **Figure S4.** Peripheral counts of CD4^+^ and CD8^+^ T cells and NK cells in each patient. (DOCX 249 kb)

Additional file 7: **Figure S5.** Flow cytometry analyses of the peripheral blood CD4low non-T cells following IT1208 treatment. (DOCX 855 kb)

Additional file 8: **Table S2.** The density of each T-cell subset in tumor foci. (DOCX 77 kb)

Additional file 9: **Figure S6.** Analyses of CD4^+^ T cells and CD4^+^ macrophages in the tumor following IT1208 treatment. (DOCX 401 kb)

Additional file 10: **Figure S7.** Transcriptomic analysis of tumors. (DOCX 556 kb)

Additional file 11: **Table S3.** Summary of TCR sequencing of PBMC samples. (DOCX 24 kb)

Additional file 12: **Table S4.** Summary of TCR sequencing of tumor biopsy samples. (DOCX 19 kb)

## Data Availability

All data generated or analyzed in this study that are relevant to the results presented in this article are included in this article and its supplementary information files (Additional file). Amplification of entire transcripts in each sample and the generation of a 3′SAGE-seq library were performed per the library construction protocol in the NCBI Gene Expression Omnibus (GEO; http:// www.ncbi.nlm.nih.gov/geo; accession GSE120028). Amplification of entire transcripts from each sample and the generation of TCR repertoire libraries were performed per the library construction protocol in the NCBI GEO; accession GSE120101. Other data that were not relevant to the results presented here are available from the corresponding author upon reasonable request.

## Abbreviations

ADCC: Antibody-dependent cellular cytotoxicity
Cmax: Maximum concentration
CTCAE: Common Terminology Criteria for Adverse Events
dLN: Draining lymph node
DLTs: Dose-limiting toxicities
Foxp3: Forkhead box P3
H&E: Hematoxylin&eosin
Inform: Image-analysis program
mAb: monoclonal antibody
MDSCs: Myeloid-derived suppressor cells
mFIHC: multiplex fluorescence Immunohistochemical
MTD: Maximum tolerated dose
PBMCs: Peripheral blood mononuclear cells
PD-1: Programmed cell death-1
pDCs: Plasmocytoid dendritic cells
PD-L1: Programmed cell death ligand-1
PFS: Progression-free survival
PR: Partial response
RD: Recommended dose
RECIST: Response Evaluation Criteria in Solid Tumors
TCR: T-cell receptor
Th2: T helper 2
Tregs: Regulatory T cells
