# Supplementary figures and images for: First-in-human phase 1 study of IT1208, a defucosylated humanized anti-CD4 depleting antibody, in patients with advanced solid tumors
Source: J Immunother Cancer. 2019 Jul 24;7:195. doi: 10.1186/s40425-019-0677-y (PMC6657210; doi:10.1186/s40425-019-0677-y)

**
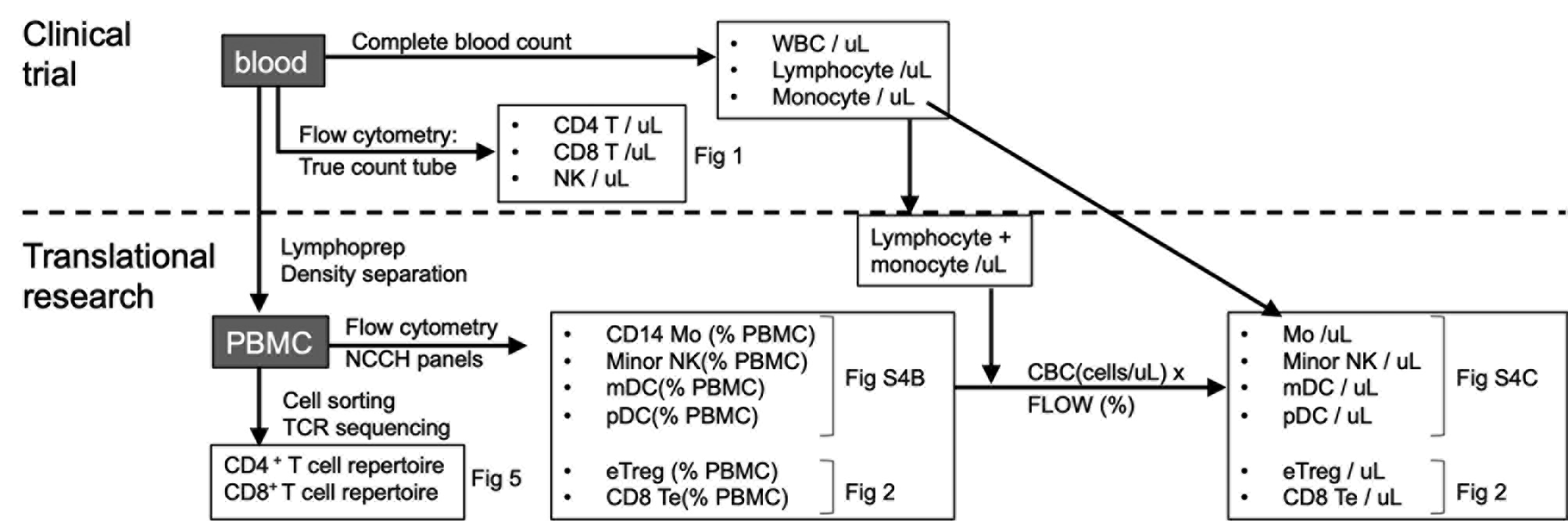
**

**Figure S1. Flowchart of blood sample analyses in clinical trial and translational research**

Supplement: Supplementary file 1 — Figure S1. Flowchart of blood sample analyses in clinical trial and translational research. (DOCX 226 kb) [file 40425_2019_677_MOESM1_ESM.docx]

**Table S2. The density of each T-cell subset in tumor foci**

**
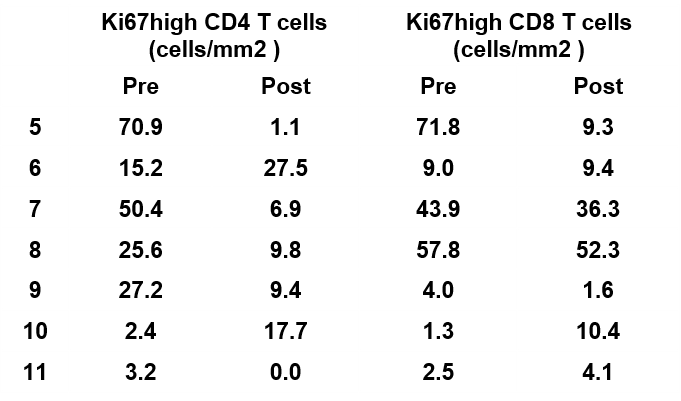

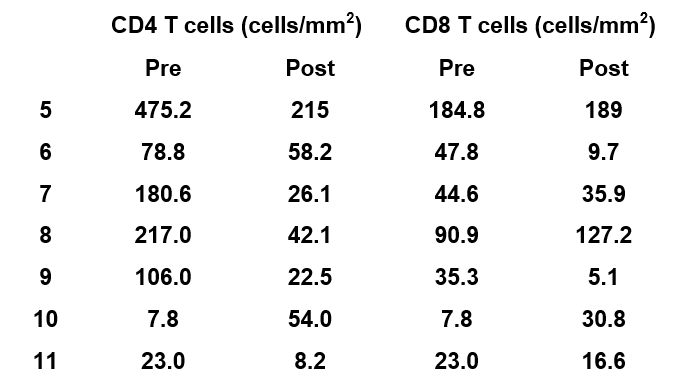

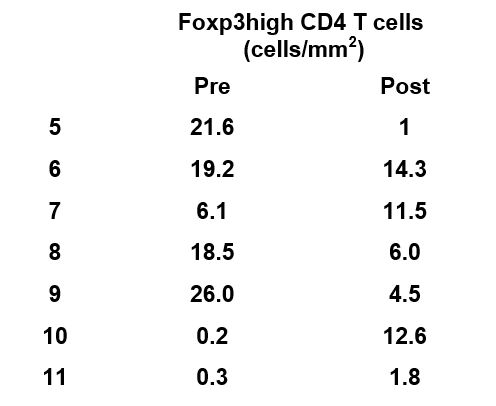
**

**Patients**

**Patients**

**Patients**

Supplement: Supplementary file 8 — Table S2. The density of each T-cell subset in tumor foci. (DOCX 77 kb) [file 40425_2019_677_MOESM8_ESM.docx]
