# Supplementary material for: First-in-human phase 1 study of IT1208, a defucosylated humanized anti-CD4 depleting antibody, in patients with advanced solid tumors
Source: J Immunother Cancer. 2019 Jul 24;7:195. doi: 10.1186/s40425-019-0677-y (PMC6657210; doi:10.1186/s40425-019-0677-y)
Supplement: Supplementary file 2 — Appendix Materials and Methods. (ZIP 358 kb) [file 40425_2019_677_MOESM2_ESM.zip › 40425_2019_677_MOESM2_ESM/40425_2019_677_MOESM13_ESM.docx]

**Table S5. Primers used for transcriptome analysis**

**

**
