# Supplementary material for: First-in-human phase 1 study of IT1208, a defucosylated humanized anti-CD4 depleting antibody, in patients with advanced solid tumors
Source: J Immunother Cancer. 2019 Jul 24;7:195. doi: 10.1186/s40425-019-0677-y (PMC6657210; doi:10.1186/s40425-019-0677-y)
Supplement: Supplementary file 2 — Appendix Materials and Methods. (ZIP 358 kb) [file 40425_2019_677_MOESM2_ESM.zip › 40425_2019_677_MOESM2_ESM/40425_2019_677_MOESM2_ESM.docx]

**Appendix Materials and Methods**

**Methodology for pharmacokinetic analysis and biomarker analysis**

**Pharmacokinetic Analysis**

For pharmacokinetic analysis in patients receiving a single administration of IT1208, blood samples were obtained before IT1208 and at 2 h after start of infusion, at end of infusion, and at 2 h after infusion on day 1 as well as on days 2, 4, 8, and 29. In patients receiving two administrations, blood samples were collected on day 1, day 8 before IT1208 as well as at 2 h after start of infusion, at end of infusion as well as on days 2, 4, 9, 11, 15, and 29.

Pharmacokinetic parameters were calculated using noncompartment analysis using the analytical approach as the intravenous infusion model generated by WinNonlin Professional version 6.4 (Certara USA, Inc., 100 Overlook Center, Suite 101, Princeton, NJ 08540, USA). The maximum concentration (*C*_max_) values were obtained from measured values. The apparent elimination half-life (*t*_1/2z_) was obtained via linear regression of three or more log-transformed data points in the terminal phase. The area under the concentration versus the time curve up to the time of the last measurable concentration data (AUC_0–72 h_ or AUC_0–28 days_) was obtained using the trapezoidal method (Linear Trapezoidal Linear Interpolation). The AUC values were extrapolated to infinity (AUC_0–inf_) using the equation AUC_0–72 h_ + C72h/*λ*, where C72h is the last measurable concentration, and *λ* is the terminal elimination rate. AUC_last_ is the AUC between end of infusion and last planned time point. The total body clearance (CL) values were calculated using the equation dose/AUC_0–inf_. Volume of distribution based on the terminal phase (*V*z) was also calculated.

**Cytokine assay**

Serum levels of interleukin-6 (IL-6), interleukin-8 (IL-8), and tumor necrosis factor-alpha (TNF-alpha) were measured using a Cytometric Bead Array (BD Biosciences) following the manufacture’s instruction. Samples were analyzed using the FACS Canto II system and FCAP Array Software 3.0 (BD Biosciences).

**Change in immune phenotypes of PBMCs**

CD4 depletion was evaluated via flow cytometry. CD45RA^+^ FoxP3^lo^ resting/naive Tregs, CD45RA^-^ FoxP3^hi^ activated/effector Tregs, and CD45RA^-^ FoxP3^lo^, non-Tregs were analyzed as described previously. Intracellular FoxP3 staining was performed using a FoxP3/Transcription Factor Staining Buffer Set (eBioscience) following the manufacturer’s instructions. Anti-CD4-peridinin-chlorophyll-protein (PerCP) (clone SK3; BD Biosciences), anti-CD25-allophycocyanin (APC) (clone 2A3; BD Biosciences), biotinylated anti-CCR4 (clone; 1G1; BD Pharmingen), Streptavidin-R-phycoerythrin (PE) (BD Biosciences), anti-CD45RA-fluorescein isothiocyanate (FITC) (clone 679.Mc7; Beckman Coulter), and anti-FoxP3-PE (clone PCH101; eBioscience) were used for phenotypic analysis of Tregs. To investigate change of CD4 low expression cells (CD14+ Monocyte, Minor NK, mDC, pDC), we used antibody panel for these cells: anti-CD3-BUV496 (clone UCHT1; BD Biosciences), anti-CD4-BV650 (clone OKT4; BioLegend), anti-CD8-APC-Cy7 (clone SK1; BioLegend), anti-CD11c-AF700 (clone B-ly6; BD Biosciences), anti-CD14-FITC (clone MΦP9; BD Biosciences), anti-CD16-BUV395 (clone 3G8; BD Bioscences), anti-CD19-PE-Cy5.5 (clone SJ25C1; eBioscience), anti-CD56-PE (clone MY31; BD Biosciences), anti-CD123-BV421 (clone 9F5; BD Biosciences) and anti-HLA-DR-ECD (clone Immu-357; Beckman Coulter).

**Multiplex Immunofluorescence (MIF) analysis**

The biopsy samples were formalin fixed and paraffin embedded (FFPE). FFPE samples were cut in 3 µm thick sections, and sections were deparaffinized and rehydrated by serial passage through changes of xylene and ethanol. Endogenous peroxidase in tissues was blocked by incubation in 0.3% hydrogen peroxide solution. Heat-induced epitope retrieval was performed in the microwave at 95 °C, 15 minutes, with appropriate antigen retrieval buffer After the blocking with 4% swine serum, slides were stained with primary antibody: rabbit IgG monoclonal anti-CD3 (clone SP7, Abcam Inc., high pH retrieval), mouse IgG1 monoclonal anti-CD4 (clone 4B, Novocastra Inc., high pH retrieval), mouse IgG2a monoclonal anti-CD8 (clone 4B11, Novocastra Inc., low pH retrieval), rabbit IgG monoclonal anti-FoxP3 (clone D6O8R, cell signaling technologies Inc., high pH retrieval), mouse IgG1 monoclonal anti-Ki67 (clone Mib-1, Agilent Dako Inc., low pH retrieval), mouse IgG1 anti-cytokeratin (clone AE1+AE3, Agilent Dako Inc., high pH retrieval), mouse IgG1 monoclonal anti-CD204 (clone SRA-E5, Transgenic Inc cat., low pH retrieval). EnVision+/HRP, Mouse (Agilent Dako Inc.) or Envision+/HRP, Rabbit (Agilent Dako Inc.) were used as the secondary antibody. Immunofluorescent signal was visualized using the Opal^TM^ 7-Color Manual IHC kit (Perkin Elmer, MA) TSA dyes 520, 540, 570, 620, 650 and 690, counterstained with Spectral DAPI. All biopsy area were imaged on the Vectra® 3.0 Automated Quantitative Pathology Imaging System, 6 Slide (Perkin Elmer, MA) and MIF data analysis (Color separation, Tissue and Cell Segmentation, and Cell Phenotyping) were performed on inForm Software v2.4 (Perkin Elmer, MA). All data of tumor or stroma area and cell number were organized by TIBCO Spotfire Data Visualization and Analytics Software (TIBCO Software Inc.).

**Transcriptomic and quantitative real-time PCR analyses**

Total RNA was extracted using a RNA easy mini extraction kit (Qiagen) following a standard protocol. Five-micrograms of total RNA were diluted with 1 mL of cell lysis buffer (100 mM Tris-HCl pH7.5, 1% LiDS, 500 mM LiCl, 10 mM EDTA, 5 mM DTT). PolyA RNAs were isolated and amplified from each RNA samples as described previously{Shichino, 2019 #1716}. Briefly, 0.5 pmol of biotin-TEG-adapter-dT25 primers was bound to 20 μL of Dynabeads M270 streptavidin (Thermo Fisher Scientific). The washed beads (20 uL) were added to each diluted RNA samples and incubated for 30 min at room temperature with gentle rotation. Beads were washed once with wash buffer A [0.1% LiDS, 10 mM Tris-HCl (pH 7.5), 150 mM LiCl, and 1 mM EDTA], two times with wash buffer B-T [10 mM Tris-HCl (pH 7.5), 150 mM LiCl, 1 mM EDTA, and 0.01% Tween-20] and once with wash buffer B [10 mM Tris-HCl (pH 7.5), 150 mM LiCl, 1 mM EDTA]. Beads were then suspended in 10 μL of RT mix 1 [1× SSIV buffer (Thermo Fisher Scientific), 2 mM dNTP, 2 M betaine (Sigma-Aldrich), 12 mM MgCl_2_, and 3.2 U/μL RNaseIn Plus (Promega)] and incubated for 90 s at 70ºC, 5 min at 35ºC, and immediately cooled on ice. RT mix 2 [10 μL; 1× SSIV buffer, 10 mM DTT (Thermo Fisher Scientific), 10 U/μL Superscript IV (Thermo Fisher Scientific), and 2 M betaine (Sigma-Aldrich)] was added, and reverse transcription was performed for 5 min at 35ºC and 15 min at 50ºC. Beads were washed once with cell lysis buffer, twice with B&W-T buffer [5 mM Tris-HCl (pH 7.5), 1 M NaCl, 0.5 mM EDTA, and 0.1% Tween-20], once with Tris-HCl (pH 8.0) and 20 μL of RNase H mix [1× first-strand buffer (Life Technologies, Carlsbad, CA, USA), 5 mM DTT, 0.6 U RNase H (Thermo Fisher Scientific)], and incubated for 20 min at 37ºC to digest reverse-transcribed mRNA. Beads were washed, 20 μL of TdT mix [50 mM Tris-HCl (pH 8.0), 100 mM KCl, 3 mM MgCl_2_, 1 mM CoCl_2_ (Roche), 0.65 mM dATP (Thermo Fisher Scientific), and 15.2 U/μL TdT (Roche)] was added on an ice-chilled aluminum rack, and polyA-tailing was performed for 3 min at 37ºC. The reaction was stopped by adding 5 μL of 0.5 M EDTA, and the enzyme was heat-inactivated by incubation for 10 min at 65ºC. Beads were washed, 20 μL of second-strand synthesis mix [1× KAPA Hifi ReadyMix (KAPA Biosystems, Wilmington, MA, USA) and 0.4 µM anchored tagging primer] was added, and second-strand synthesis was performed according to the following program: 95ºC for 2 min, 98ºC for 20 s, 50ºC for 2 min, 72ºC for 7 min, and hold at 4ºC. Beads were washed, and 1/4 of the beads were used for the first round of whole-transcript amplification (WTA) in 25 μL of first-round WTA mix [1× KAPA Hifi ReadyMix (KAPA Biosystems), 0.4 µM anchored tagging primer, and 0.4 µM 3′ WTA primer] using the following program: 95ºC for 3 min, five cycles of 98ºC for 20 s, 65ºC for 15 s, and 72ºC for 7 min, followed by 72ºC for 5 min and a hold at 4ºC. PCR products were purified twice with 0.6× AmPure XP beads (Beckman Coulter) and eluted with 23 μL of nuclease-free water. Second-round WTA mix [27 μL; 1× KAPA Hifi ReadyMix (KAPA Biosystems), 0.614 µM 5′ WTA primer, and 0.614 µM Biotin-TEG-3′ WTA primer] was added, and the second round of WTA was performed using the following program: 95ºC for 3 min, nine cycles of 98ºC for 20 s, 65ºC for 15 s, and 72ºC for 7 min, followed by 72ºC for 5 min and a hold at 4ºC. PCR products were purified twice with 0.6× AmPure XP beads and eluted with 25 μL of Tris-HCl (pH 8.0). Amplified whole transcripts were quantified using a Nanodrop 1000 (Thermo Fisher Scientific), and size distribution was analyzed by agarose electrophoresis and SYBR Gold staining (Thermo Fisher Scientific).

3′SAGE-seq libraries were constructed as described previously{Shichino, 2019 #1716}. Briefly, 150 ng of the whole-transcript library was restriction digested with NlaIII (New England Biolabs, Ipswich, MA, USA) for 2 h at 37ºC, and biotinylated 3′-tail transcripts were immobilized on Dynabead M-280 streptavidin beads (Thermo Fisher Scientific). Beads were washed, and 10 pmol of the CS1-EcoP15I-NlaIII adapter was ligated using a DNA ligation kit (Mighty Mix; Takara) for 30 min at 16ºC. Beads were washed three times with B&W-T buffer, once with 10 mM Tris-HCl (pH 8.0), suspended in 200 μL of EcoP15I digestion mix [1× NEBuffer 3.1, 1 mM ATP, and 0.2 U EcoP15I (New England Biolabs)], and digested for 16 h at 37ºC in a tightly sealed screw-cap tube with gentle rotation. Supernatant was purified using a Nucleospin Gel&PCR clean-up kit (Takara) and eluted twice with 12.5 μL of nuclease-free water. End-repair/polyA-tailing/ligation reactions were performed using NEBNext Ultra II modules (New England Biolabs) and 1.875 pmol of CS2-adapter according to manufacturer instructions. Reaction products were purified using a Qiagen MinElute Column (Qiagen, Hilden, Germany) and eluted with 13 μL of nuclease-free water. Barcoding mix [14.25 μL; 1× KAPA Hifi ReadyMix (KAPA Biosystems), 0.614 µM IonA-BC[N]-CS1-primer (Additional file13: Table S5), and 0.614 µM Ion-trP1-CS2 primer] was added into 10.75 μL of elutant, and PCR enrichment was performed using the following program: 98ºC for 45 s, nine cycles of 98ºC for 15 s, 65ºC for 30 s, and 72ºC for 90 s, followed by 72ºC for 1 min and a hold at 4ºC. Reaction products were purified using double-size selection of AmPure XP beads (0.8× → 0.8×) and eluted with 10 μL of Tris-HCl (pH 8.0). The size distribution of each library was analyzed using an Agilent DNA high-sensitivity kit (Agilent Technologies, Santa Clara, CA, USA), and library concentration was quantified using a KAPA library quantification kit for Ion Torrent (KAPA Biosystems). Primer sequences are shown in Table S3. All oligos for transcriptome data analysis were HPLC purified and purchased from Integrated DNA Technologies (Coralville, IA, USA) or Sigma-Genosys (The Woodlands, TX, USA). Sequencing was performed using an Ion Hi-Q Chef kit, an Ion PI v3 Chip kit, and an Ion Proton Sequencer (Thermo Fisher Scientific) following the manufacturer’s instructions, except that the input library concentration was 100 pM. Raw data obtained from these experiments have been deposited in the NCBI GEO; accession GSE120028. qPCR analysis was performed using a Thunderbird SYBR qPCR Mix (Toyobo, Osaka, Japan) on a QuantStudio6 real-time PCR system (Applied Biosystems, Foster City, CA, USA). Sequences of the primers for *RPS3* are as follows: Forward 5′’-CCCGCGAGCCACTTCCTTTC-3′, Reverse 5′-TGTCCTGGTTGGTGTAACTCG-3′. TaqMan Gene Expression Assay probes for *GZMB* (Hs00188051_m1), *IFNG* (Hs00989291_m1), *PDCD1* (Hs01550088_m1), *CD8A* (Hs00233520_m1), *FOXP3* (Hs01085834_m1), *CD274* (Hs00204257_m1), and *CD4* (Hs01058407_m1) were purchased from Applied Biosystems. The expression levels of all target mRNAs were normalized against the expression level of *RPS3* in each sample.

**Transcriptome data analysis**

Adapter trimming and quality filtering of sequencing data were performed using Trimommatic-v0.36^1^ and PRINSEQ-0.20.4^2^. Filtered reads were mapped to Refseq hg38 using Bowtie2-2.2.5^3^ with the following parameters: -t -p 11 -N 1 -D 200 -R 20 -L 20 -i S,1,0.50 --norc. Reads that were not mapped to NlaIII sites were removed, and tag numbers of each gene were quantified as the expression level of each gene. Between-sample normalization was performed against raw count data using Microsoft R open 3.5.0 (https://mran.microsoft.com/open/) and TCC package (DDD-D method)^4^. Genes with fold change ≥2 and maximum expression ≥30 were identified as differentially expressed genes. Co-expressed gene modules among differentially expressed genes were detected using the WGCNA package^5^ in Microsoft R open 3.5.0. Log_2_ fold-change values between pre- and post-treatment in the same patients were used as input for the WGCNA package. For WGCNA, the power value was 27, the merge threshold value was 0.25, the threshold value for the output of co-expression interactions was 0.25, and other calculation settings were set to defaults. The genes in tumor reduction-associated gene modules (M12, M14–M17, M20, M23, and M24) detected using WGCNA were further clustered into positively and negatively correlated gene groups using the CLICK method^97^ and visualized using Microsoft R open 3.5.0. Functional analysis of the tumor reduction-associated gene module groups was performed using Cytoscape 3.3.0 with ClueGO plugin (v2.4.3)^6,7^. Significantly enriched GO terms^8^ (GO biological process, GO levels: 3–8) and Kyoto Encyclopedia of Genes and Genomes (KEGG) pathway terms^9^ in gene modules (M12, M14–M17, M20, M23, and M24) were explored, grouped, and a network terms were constructed based on the overlap of their elements (kappa score = 0.4). Leading terms within each group were defined as the most significantly enriched term in each group. We used versions of the GO term database and KEGG pathway term database that were current on Sep 21, 2017.

**T-cell isolation from PBMCs and biopsy specimens**

T cells were enriched from the PBMCs using the Pan T-Cell Isolation Kit, human (Milteny Biotec Inc., Bergisch Gladbach, Germany). Cells were stained with CD8-FITC (clone BW135/80, Miltenyi Biotec), CD3-PE (clone UCHT1, TONBO Biosciences, San Diego, CA, USA), CD4-PerCP-Cy5.5 (clone SK3, TONBO Biosciences), and Ghost Dye Red780 (TONBO Biosciences), and then CD3^+^ CD4^+^ and CD3^+^ CD8^+^ T cells were purified using a FACS Aria II with Diva software (BD Biosciences). Purity of sorted cells was routinely more than 99%. After washing, the sorted cells were lysed in 1 mL of cell lysis buffer and used for whole transcript amplification.

**TCR repertoire sequencing**

CD4^+^ and CD8^+^ T cells were purified by cell sorting at days 1 and 22 and were lysed in lysis buffer. Tumor biopsies were collected before and at day 29 after treatment and Total RNA was extracted as described in “Transcriptomic and quantitative real-time PCR analyses”. Primers used for TCR repertoire sequencing are presented as additional file14: Table S6. PolyA RNAs were isolated and amplified from T cell lysate or total RNA samples of the tumor as described in “Transcriptomic and quantitative real-time PCR analyses”. To amplify the TCR cDNA containing complementarity determining region 3 (CDR3), nested PCR of the TCR locus was performed as follows. The first PCR mixture comprised 0.4 μL of 10 μM primer mix (5′ WTA and Trbc_ex), 4.6 μL of template, and 5 μL of KAPA Hifi Hotstart ReadyMix (KAPA Biosystems, Wilmington, MA, USA, #KK2602). The thermal cycling conditions were programmed as follows: denaturation at 95°C for 3 min, 10 cycles of denaturation for 20 sec at 98°C, annealing for 15 sec at 58°C and extension for 30 sec at 72°C, followed by final extension at 72°C for 2 min. Next, 10 μL of the first-PCR products was used for purification with an Agencort AM Pure XP kit (Bexkman-Coulter, CA, USA, #A63881) at a 0.7:1 ratio of beads to sample, and eluted with 12 μL of DW. The second PCR mixture consisted of 1.25 μL of 10 μM primer mix (5′ WTA and Biotinylated Trbc_in), 11.25 μL of template and 12.5 μL of KAPA Hifi Hotstart ReadyMix. The thermal cycling conditions were same as the first PCR except the cycle number; 13 cycles. Next, 25 μL of the second-PCR products were purified using Agencort AM Pure XP kit (Bexkman-Coulter) at a 0.8:1 ratio of beads to sample, and eluted in 15 μL of DW. The purified PCR products were sheared randomly using NEBNext dsDNA fragmentase (New England Biolabs, #M0348). The fragmentation reaction mix consisted of 6 μL of DW, 2 μL of 10X Fragmentase Reaction Buffer v2, 10 μL of PCR product, and 2 μL of fragmentase. The fragmentation reaction was incubated at 37°C for 30 min, and then 5 μL of 0.5 M EDTA was added to stop the reaction on ice. The sheared PCR product was then purified and subjected to size selection using the Agencort AM Pure XP kit (Bexkman-Coulter) at a 0.8:1 ratio of beads to sample to remove large fragments, a 0.8:1 ratio of beads to sample to remove the smaller fragments, and eluted with 20 μL of Tris-HCl (pH 8.0). To capture the TCR cDNA containing the end of Constant region, the purified PCR products were incubated with 10 μL of Dynabeads M-270 Streptavidin (Thermo Fisher Scientific, MA, USA, #DB65305) for 30 min at room temperature (r.t.), washed 3 times with B&W-T buffer [5 mM Tris-HCl (pH 7.5), 1 M NaCl, 0.5 mM EDTA, and 0.1% Tween-20], and once with Tris-HCl (pH 8.0), and DW. The captured TCR cDNA was repaired using NEBNext Ultra II End Repair/ dA-Tailing Module (New England Biolabs, # E7546L). The repair reaction mix contained 1.2 μL of reaction buffer, 8.3 μL of beads re-suspended with DW, and 0.5 μL of enzyme mix. The repair reaction was incubated at 20°C for 30 min, then washed once with lysis buffer, 3 times with B&W-T buffer, and once with Tris-HCl (pH 8.0). The repaired TCR cDNA was attached to the sequencing adaptor using the DNA Ligation Kit ＜Mighty Mix＞ (TaKaRa, Shiga, Japan, #6023). The ligation reaction mix consisted of 1 μL of 10 μM P1 adaptor, 6.5 μL of beads re-suspended in Tris-HCl (pH 8.0), and 15 μL of enzyme mix. The ligation reaction was incubated at 16°C for 60 min using a thermal cycler with the cover open, washed once with lysis buffer, 3 times with B&W-T buffer, and once with Tris-HCl (pH 8.0). The third PCR were carried out using barcoded primers to enrich the TCR cDNA flanked with sequencing adapters. The third PCR mixture consisted of 0.35 μL of 10 μM trP1 primer, 1 μL of 3.5 μM IonA-BC-Trbc primer, 3.65 μL of beads resuspended in DW and 5 μL of KAPA Hifi Hotstart ReadyMix. The thermal cycling conditions were same as the first PCR except the cycle number; 12 cycles. The PCR product was purified and subjected to size selection using Agencort AM Pure XP kit (Bexkman-Coulter,) at a 0.75:1 ratio of beads to sample to remove large fragments, a 0.65:1 ratio of beads to sample to remove smaller fragments, and eluted with 20 μL of Tris-HCl (pH 8.0). Amplified TCR libraries were quantified using a KAPA Library Quantification Kit (KAPA Biosystems, Wilmington, MA, USA, #KK4827) and size distribution was analyzed by agarose electrophoresis and SYBR Gold staining (Thermo Fisher Scientific, #S11494). Primer sequences are shown in Appendix Table A3.

Final TCR libraries, whose lengths were 200–300 base pairs, were pooled and sequenced using an Ion Hi-Q Chef kit, an Ion PI v3 Chip kit, and an Ion Proton Sequencer or Ion S5 Sequencer (Thermo Fisher Scientific) according to the manufacturer’s instructions, except the input library concentration (100 pM) and flow number (500). Raw data from these experiments have been deposited in the NCBI GEO; accession GSE120101.

**TCR repertoire data analyses**

Adapter sequences and low-quality reads were trimmed using cutadapt-v1.11^10^ and PRINSEQ-0.20.4^2^. Filtered reads were processed using “analyze amplicon” command of MiXCR-3.0.2^11^ with following high-level options: -s = hsa, -starting-material = rna, -5-end = no-v-primers, -3-end = c-primers, -adapters = no-adapters, -receptor-type = trb, -region-of-interest = CDR3. In alignment to ImMunoGeneTics (IMGT) reference human TCR V/D/J sequences^12^, we used the following parameters: -OvParameters.geneFeatureToAlign = VTranscript -OvjAlignmentOrder = JThenV. In assembling identical sequences into clones with PCR and sequencing error correlations, we used the following parameters: -ObadQualityThreshold=10, -OseparateByV=true. Only clones with productive CDR3 sequences were analyzed using VDJtools (ver 1.2.1.)^13^. Then, the sequencing coverage of samples, which was defined as the ratio of total reads to the starting number of T cells, was normalized to ×5 using the “DownSample” command in VDJtools. After coverage normalization, clones with lead count less than sequencing coverage were excluded as sequencing noise. For PBMC sample, we identified clones shared between CD4^+^ and CD8^+^ T cell repertoire as contamination, and separated these shared clones into CD4^+^ or CD8^+^ repertoire by their frequency in CD4^+^ or CD8^+^, because these shared clones were very few and their frequencies were biased to either CD4^+^ or CD8^+^ (Data not shown). The processed data have been deposited at the NCBI GEO; accession GSE120101. The sequence performance of PBMC and tumor samples are summarized in additional file11: Table S3 and additional file 12: Table S4.

The T-cell clones were determined as TCR reads with the same TCR variable (V) segment, joining (J) segment, and CDR3 nucleotide sequence. The similarity in TCR repertoires pre- and post-IT1208 treatment was calculated as R similarity index; the Pearson product-moment correlation coefficient of frequencies of overlapping clones between two time points. The clonality of the TCR repertoire was calculated as 1 - Pielou index, which was calculated using the formula 1 − ∑𝑛𝑖=1 𝑝𝑖 log𝑒(𝑝𝑖)/ log𝑒(𝑛), where 𝑝𝑖 is the frequency of clone *i* for a sample with *n* unique clones. Of note, this metric is normalized to the number of unique clones and ranges from 0 to 1. Statistical analyses were performed using GraphPad Prism (ver7) software (GraphPad Software, La Jolla, CA, USA).

**References**

1. Bolger AM, Lohse M, Usadel B: Trimmomatic: a flexible trimmer for Illumina sequence data. Bioinformatics 30:2114-20, 2014
2. Schmieder R, Edwards R: Quality control and preprocessing of metagenomic datasets. Bioinformatics 27:863-4, 2011
3. Langmead B, Salzberg SL: Fast gapped-read alignment with Bowtie 2. Nat Methods 9:357-9, 2012
4. Tang M, Sun J, Shimizu K, et al: Evaluation of methods for differential expression analysis on multi-group RNA-seq count data. BMC Bioinformatics 16:361, 2015
5. Langfelder P, Horvath S: WGCNA: an R package for weighted correlation network analysis. BMC Bioinformatics 9:559, 2008
6. Bindea G, Mlecnik B, Hackl H, et al: ClueGO: a Cytoscape plug-in to decipher functionally grouped gene ontology and pathway annotation networks. Bioinformatics 25:1091-3, 2009
7. Saito R, Smoot ME, Ono K, et al: A travel guide to Cytoscape plugins. Nat Methods 9:1069-76, 2012
8. Gene Ontology C: Gene Ontology Consortium: going forward. Nucleic Acids Res 43:D1049-56, 2015
9. Kanehisa M, Goto S: KEGG: kyoto encyclopedia of genes and genomes. Nucleic Acids Res 28:27-30, 2000
10. Martin M: Cutadapt removes adapter sequences from high-throughput sequencing reads. EMBnet.journal 17:10-12, 2011
11. Bolotin DA, Poslavsky S, Mitrophanov I, et al: MiXCR: software for comprehensive adaptive immunity profiling. Nat Methods 12:380-1, 2015
12. Giudicelli V, Duroux P, Ginestoux C, et al: IMGT/LIGM-DB, the IMGT comprehensive database of immunoglobulin and T cell receptor nucleotide sequences. Nucleic Acids Res 34:D781-4, 2006
13. Shugay M, Bagaev DV, Turchaninova MA, et al: VDJtools: Unifying Post-analysis of T Cell Receptor Repertoires. PLoS Comput Biol 11:e1004503, 2015
