# Supplementary material for: First-in-human phase 1 study of IT1208, a defucosylated humanized anti-CD4 depleting antibody, in patients with advanced solid tumors
Source: J Immunother Cancer. 2019 Jul 24;7:195. doi: 10.1186/s40425-019-0677-y (PMC6657210; doi:10.1186/s40425-019-0677-y)
Supplement: Supplementary file 3 — Table S1. IT1208-related adverse events. (DOCX 13 kb) [file 40425_2019_677_MOESM3_ESM.docx]

**Table S1. IT1208-related adverse events**

| **Adverse event** | **Dose level 1**  **(*n* = 4)** | | | | **Dose level 2**  **(*n* = 7)** | | | |
| --- | --- | --- | --- | --- | --- | --- | --- | --- |
| **Grade** | **1** | **2** | **3** | **4** | **1** | **2** | **3** | **4** |
| **Infusion-related reactions** | **4** | **0** | **0** | **0** | **1** | **6** | **0** | **0** |
| **Fever** | **4** | **0** | **0** | **0** | **0** | **4** | **0** | **0** |
| **Diarrhea** | **0** | **0** | **0** | **0** | **1** | **1** | **0** | **0** |
| **Nausea** | **0** | **0** | **0** | **0** | **0** | **3** | **0** | **0** |
| **Vomiting** | **0** | **0** | **0** | **0** | **0** | **3** | **0** | **0** |
| **Tumor pain** | **0** | **0** | **0** | **0** | **0** | **1** | **0** | **0** |
| **Decreased oxygen saturation** | **0** | **0** | **0** | **0** | **0** | **1** | **0** | **0** |
| **Hypotension** | **0** | **0** | **0** | **0** | **0** | **1** | **0** | **0** |
| **Flushing** | **0** | **0** | **0** | **0** | **0** | **1** | **0** | **0** |
| **Chills** | **0** | **0** | **0** | **0** | **0** | **6** | **0** | **0** |
