# Supplementary material for: First-in-human phase 1 study of IT1208, a defucosylated humanized anti-CD4 depleting antibody, in patients with advanced solid tumors
Source: J Immunother Cancer. 2019 Jul 24;7:195. doi: 10.1186/s40425-019-0677-y (PMC6657210; doi:10.1186/s40425-019-0677-y)
Supplement: Supplementary file 4 — Figure S2. Pharmacokinetics of IT1208 and serum level of cytokines. (DOCX 128 kb) [file 40425_2019_677_MOESM4_ESM.docx]

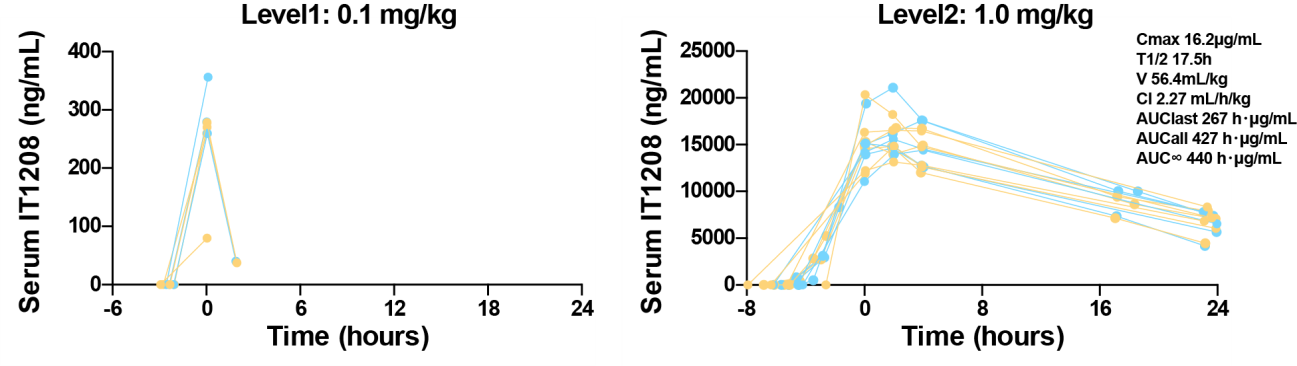


**Figure S2. Pharmacokinetics of IT1208 and serum level of cytokines**

The maximum plasma concentration (*C*_max_) of IT1208 at 0.1 mg/kg after one or two administrations was 0.255 ± 0.0838 µg/mL (average ± standard deviation) and decreased to less than the detection threshold (0.03 µg/mL) at 4 h after the end of infusion. Yellow lines indicate plasma concentrations at first administration and blue lines indicate concentrations at second administrations. AUC_last_ and AUC_0–72 h_ was 0.130 ± 0.155 and 0.280 ± 0.0941 µg·h/mL. *C*_max_ of IT1208 at 1 mg/kg after one or two administrations was 16.2 ± 2.32 µg/mL and decreased to less than the detection threshold at 72 h after the end of infusion with *t*_1/2z_ of 17.5 ± 2.54 h. Exposure was dose proportional and no accumulation of IT1208 was observed at either dose level before the second infusion on day 8. AUC_last_, AUC_0–72 h_, and AUC_0–inf_ was 267 ± 33.5, 427 ± 62.6, and 440 ± 75.9 µg·h/mL. Total body clearances and volume of distribution was 2.27 ± 0.422 mL/h/kg and 56.4 ± 7.61 mL/kg.
