# Supplementary material for: First-in-human phase 1 study of IT1208, a defucosylated humanized anti-CD4 depleting antibody, in patients with advanced solid tumors
Source: J Immunother Cancer. 2019 Jul 24;7:195. doi: 10.1186/s40425-019-0677-y (PMC6657210; doi:10.1186/s40425-019-0677-y)
Supplement: Supplementary file 5 — Figure S3. Serum level of cytokines. (DOCX 99 kb) [file 40425_2019_677_MOESM5_ESM.docx]

**
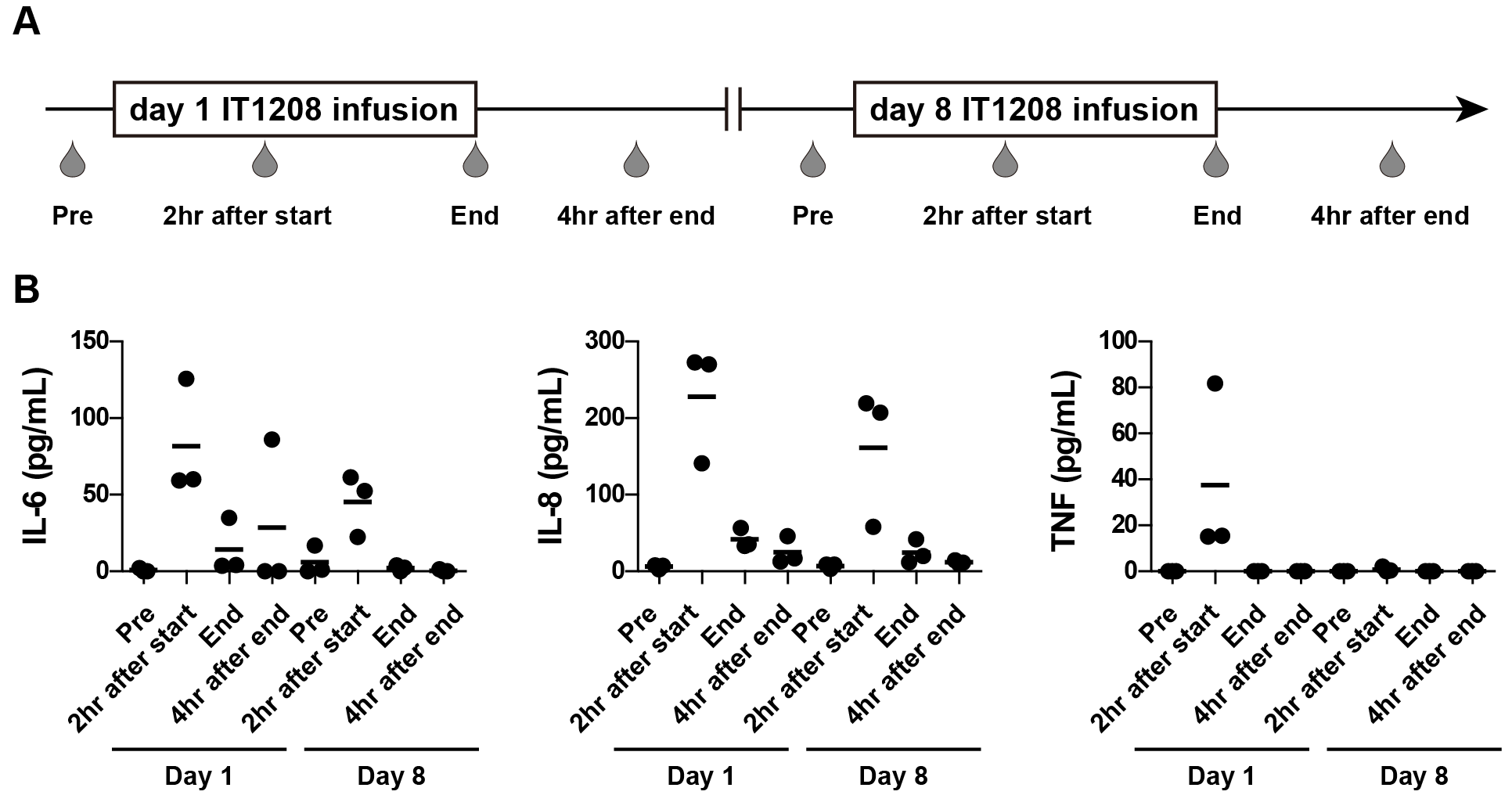
**

**Figure S3. Serum level of cytokines**

**A.** Serum samples were prepared from patients receiving two doses of 1.0 mg/kg IT1208 infusion before (Pre), 2 h after initial IT1208 infusion (2 h after start), end of infusion (end) and 2 h after end of infusion (4 h after end) at days 1 and 8. **B.** Serum levels of IL-6, IL-8, TNF-alpha were determined using a Cytometric Bead Array with FACS Canto II system and FCAP Array Software 3.0.
