# Supplementary material for: First-in-human phase 1 study of IT1208, a defucosylated humanized anti-CD4 depleting antibody, in patients with advanced solid tumors
Source: J Immunother Cancer. 2019 Jul 24;7:195. doi: 10.1186/s40425-019-0677-y (PMC6657210; doi:10.1186/s40425-019-0677-y)
Supplement: Supplementary file 6 — Figure S4. Peripheral counts of CD4+ and CD8+ T cells and NK cells in each patient. (DOCX 249 kb) [file 40425_2019_677_MOESM6_ESM.docx]

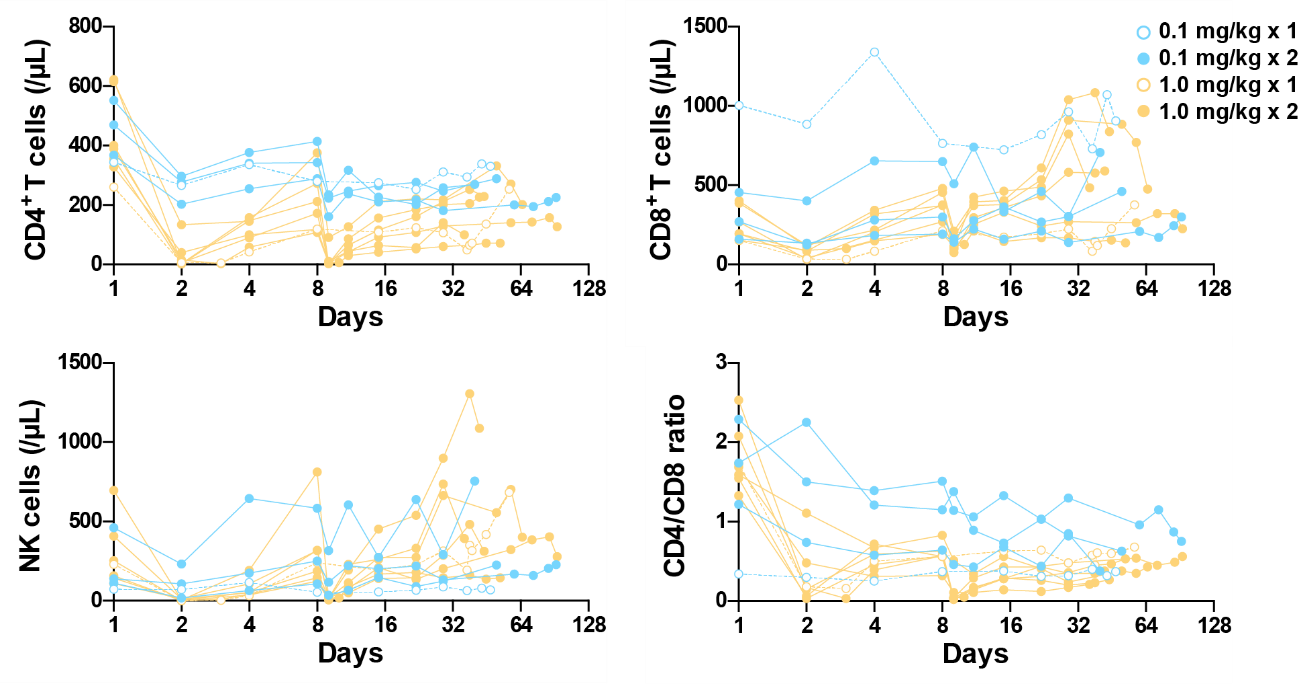


**Figure S4. Peripheral counts of CD4^+^ and CD8^+^ T cells and NK cells in each patient**

Peripheral counts of CD4^+^ and CD8^+^ T cells and NK cells in each patient and each dose level. Decreased CD4^+^ T-cell count in PBMCs due to IT1208 was observed in all patients, especially in patients receiving 1.0 mg/kg. CD8^+^ T-cell counts were also decreased immediately after IT1208 administration but then increased until day 29 and surpassed the baseline counts in most patients, which resulted in remarkably decreased CD4/8 ratios.
