# Supplementary material for: First-in-human phase 1 study of IT1208, a defucosylated humanized anti-CD4 depleting antibody, in patients with advanced solid tumors
Source: J Immunother Cancer. 2019 Jul 24;7:195. doi: 10.1186/s40425-019-0677-y (PMC6657210; doi:10.1186/s40425-019-0677-y)
Supplement: Supplementary file 7 — Figure S5. Flow cytometry analyses of the peripheral blood CD4low non-T cells following IT1208 treatment. (DOCX 855 kb) [file 40425_2019_677_MOESM7_ESM.docx]

**Figure S5. Flow cytometry analyses of the peripheral blood CD4^low^ non-T cells** **following IT1208 treatment**

**A.** Representative FCM profiles showing CD4^low^ cells among CD3^-^ PBMCs. Kinetics of the frequency **(B**) and cell number / uL blood (**C**) of CD14^+^ monocytes, minor NK cells, mDCs and pDCs in the PBMCs in each patient. P values in the table represent comparison between before and after IT1208 treatment (Wilcoxon matched-pairs signed rank test).
