# Supplementary material for: First-in-human phase 1 study of IT1208, a defucosylated humanized anti-CD4 depleting antibody, in patients with advanced solid tumors
Source: J Immunother Cancer. 2019 Jul 24;7:195. doi: 10.1186/s40425-019-0677-y (PMC6657210; doi:10.1186/s40425-019-0677-y)
Supplement: Supplementary file 9 — Figure S6. Analyses of CD4+ T cells and CD4+ macrophages in the tumor following IT1208 treatment. (DOCX 401 kb) [file 40425_2019_677_MOESM9_ESM.docx]

**Figure S6. Analyses of CD4^+^ T cells and CD4^+^ macrophages in the tumor following IT1208 treatment**

**A.** Biopsy specimen from patient 10 after treatment were stained with antibodies against CD4, CD3, CD204, and CK. Three-color images (CD4/CD3/DAPI and CD4/CD204/DAPI) were split from a five-color image to make it easy to recognize CD3^hi^ CD4^+^ T cells and CD204^hi^ CD4^+^ macrophages. Arrow heads represent double positive cells. **B.** CD3^hi^ CD4^+^ or CD204^hi^ CD4^+^ area in the CK5^+^ tumor area was quantified by an image-analysis program. Each dot represents one specimen from a patient. The result from biopsy specimens from six patients receiving two doses of 1.0 mg /kg was shown. P values in the figure represent comparison between pre and post IT1208 treatment (Wilcoxon matched-pairs signed rank test).
