# Supplementary material for: First-in-human phase 1 study of IT1208, a defucosylated humanized anti-CD4 depleting antibody, in patients with advanced solid tumors
Source: J Immunother Cancer. 2019 Jul 24;7:195. doi: 10.1186/s40425-019-0677-y (PMC6657210; doi:10.1186/s40425-019-0677-y)
Supplement: Supplementary file 10 — Figure S7. Transcriptomic analysis of tumors. (DOCX 556 kb) [file 40425_2019_677_MOESM10_ESM.docx]

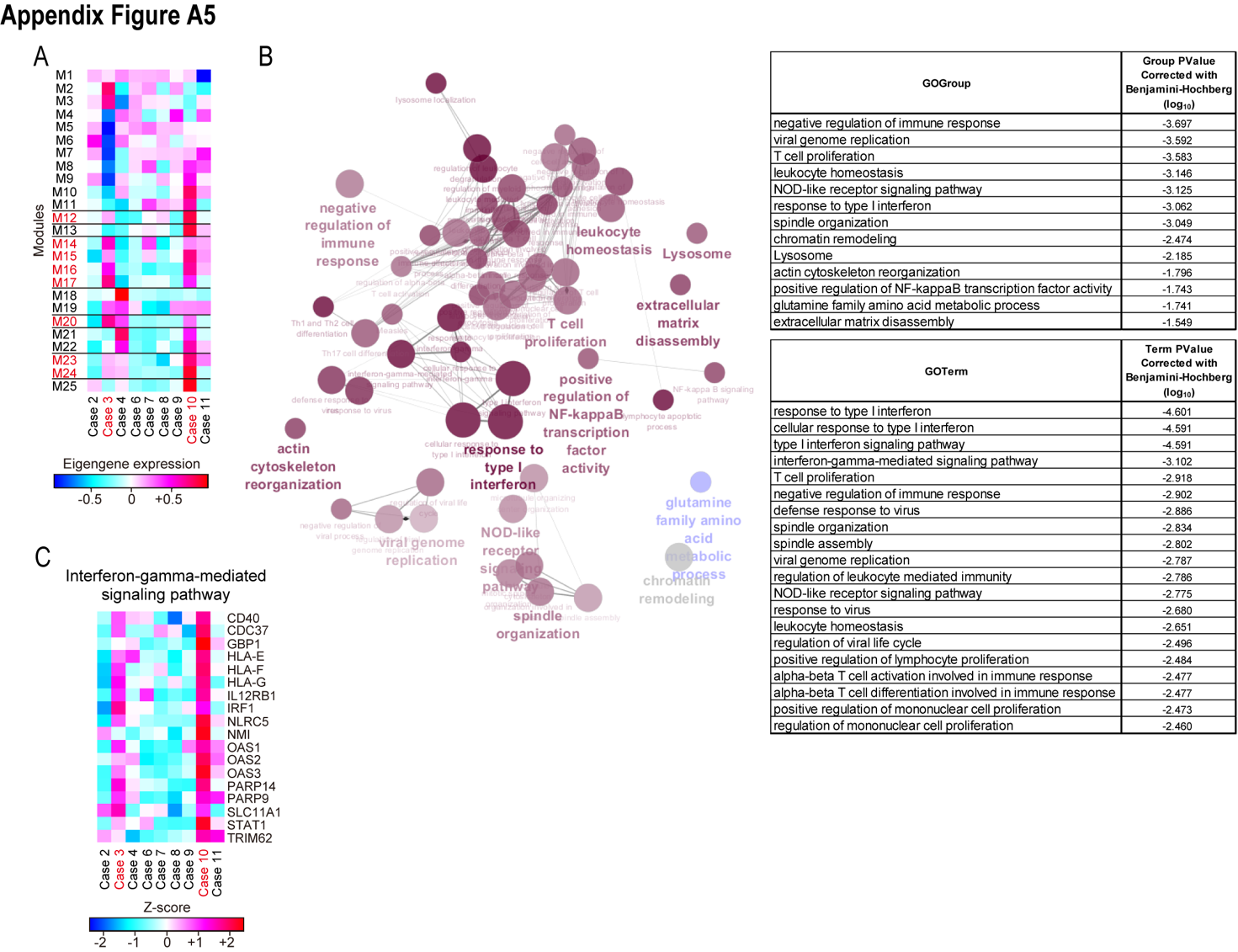
 **Figure S7. Transcriptomic analysis of tumors.**

**A.** Heat map representation of eigengenes of gene modules identified using the weighted co-expression network analysis. Each column represents cases, whereas each row represents an individual eigengene. **B.** Network of significantly enriched functional terms of gene modules (M12, M14–M17, M20, M23, and M24). Biological events associated with the modules were explored, and network terms were clustered using Cytoscape 3.3.0 with ClueGO plugins. Each node and its size represent a functional term and enrichment significance, respectively. Brown nodes represent functional terms for which genes upregulated in cases 3 and 10 comprised over 60% of all genes. Blue nodes represent functional terms for which genes downregulated in cases 3 and 10 comprised over 60% of all genes. Statistical significance was calculated for each term using the two-sided hypergeometric test with the Benjamini–Hochberg correction. Group leading terms, top 20 enriched terms, and associated *P*-values are shown. **C.** Heat map of the changes in gene expression in the tumor biopsies following IT1208 treatment. The genes associated with interferon-gamma-mediated signaling
