# Supplementary material for: First-in-human phase 1 study of IT1208, a defucosylated humanized anti-CD4 depleting antibody, in patients with advanced solid tumors
Source: J Immunother Cancer. 2019 Jul 24;7:195. doi: 10.1186/s40425-019-0677-y (PMC6657210; doi:10.1186/s40425-019-0677-y)
Supplement: Supplementary file 11 — Table S3. Summary of TCR sequencing of PBMC samples. (DOCX 24 kb) [file 40425_2019_677_MOESM11_ESM.docx]

**Table S3. Summary of TCR sequencing of PBMC samples**

A: Sequencing coverage, defined as the ratio of total reads to the start cell count, was normalized to ×5.

B: Clonality was calcuated as the 1 - Pielou index.
